# The metazoan history of the COE transcription factors. Selection of a variant HLH motif by mandatory inclusion of a duplicated exon in vertebrates

**DOI:** 10.1186/1471-2148-8-131

**Published:** 2008-05-02

**Authors:** Virginie Daburon, Sébastien Mella, Jean-Louis Plouhinec, Sylvie Mazan, Michèle Crozatier, Alain Vincent

**Affiliations:** 1 Centre de Biologie du Développement, UMR 5547 and IFR 109 CNRS/UPS, 118 route de Narbonne 31062 Toulouse cedex 4, France; 2 MRC Human Genetics Unit, Western General Hospital, Edinburgh EH4 2XU, UK; 3 Développement et Evolution des vertébrés, UMR 6218, 3b rue de la Ferollerie 45071 ORLEANS cedex 2, France; 4 Howard Hughes Medical Institute and Department of Biological Chemistry, University of California, Los Angeles, CA 90095-1662, USA

## Abstract

**Background:**

The increasing number of available genomic sequences makes it now possible to study the evolutionary history of specific genes or gene families. Transcription factors (TFs) involved in regulation of gene-specific expression are key players in the evolution of metazoan development. The low complexity COE (Collier/Olfactory-1/Early B-Cell Factor) family of transcription factors constitutes a well-suited paradigm for studying evolution of TF structure and function, including the specific question of protein modularity. Here, we compare the structure of *coe *genes within the metazoan kingdom and report on the mechanism behind a vertebrate-specific exon duplication.

**Results:**

COE proteins display a modular organisation, with three highly conserved domains : a COE-specific DNA-binding domain (DBD), an Immunoglobulin/Plexin/transcription (IPT) domain and an atypical Helix-Loop-Helix (HLH) motif. Comparison of the splice structure of *coe *genes between cnidariae and bilateriae shows that the ancestral COE DBD was built from 7 separate exons, with no evidence for exon shuffling with other metazoan gene families. It also confirms the presence of an ancestral H1LH2 motif present in all COE proteins which partly overlaps the repeated H2d-H2a motif first identified in rodent EBF. Electrophoretic Mobility Shift Assays show that formation of COE dimers is mediated by this ancestral motif. The H2d-H2a α-helical repetition appears to be a vertebrate characteristic that originated from a tandem exon duplication having taken place prior to the splitting between gnathostomes and cyclostomes. We put-forward a two-step model for the inclusion of this exon in the vertebrate transcripts.

**Conclusion:**

Three main features in the history of the *coe *gene family can be inferred from these analyses: (i) each conserved domain of the ancestral *coe *gene was built from multiple exons and the same scattered structure has been maintained throughout metazoan evolution. (ii) There exists a single *coe *gene copy per metazoan genome except in vertebrates. The H2a-H2d duplication that is specific to vertebrate proteins provides an example of a novel vertebrate characteristic, which may have been fixed early in the gnathostome lineage. (iii) This duplication provides an interesting example of counter-selection of alternative splicing.

## Background

Thanks to the increasing number of available genomic sequences, it has become possible to study the evolutionary history of specific genes or gene families in relation to their co-option in innovations that have punctuated the evolutionary diversification of metazoans. Transcription factors (TFs) involved in regulation of gene-specific expression are key players in the evolution of development. The COE family of transcription factors takes its name from the founding members of the family, Collier (Col) and Olfactory-1/Early-B-Cell Factor (Olf-1/EBF) isolated from *Drosophila *and rodents, respectively [1-3]. While there was no evidence for *coe *genes in either fungi, plants, or any of the various phyla of protozoans, identification of a cnidarian *coe *gene, *Nvcoe*, in the anthozoan sea anemone *Nemostella vectensis*, suggested that COE proteins have appeared with metazoa [4], a conclusion strengthened by the identification of COE members both in other cnidaria and porifera [5]. While up to 4 *ebf *paralogs have been identified in vertebrates [6-8], a single *coe *member has been identified in all the other animals for which genome sequences have become available, suggesting that expansion of the *coe *gene family only occurred at the origin of vertebrates.

Expression profiles of *coe *genes in embryos from various protostomes and deuterostomes and *N. vectensis *have revealed a common feature, namely, an expression in subsets of sensory neurons [4,9-14]. This feature raised the possibility that one ancestral role of COE proteins was to participate in the specification of specialised sensory cells and the ontogeny of an elaborate nervous system [12]. However, genetic analyses performed in mice and, more recently, *Drosophila *raised the possibility that another ancestral function of COE proteins could have been in development of cellular immunity [15,16]. The diversity of COE protein functions strikingly contrasts with the high degree of primary sequence conservation and lack of expansion of this family of TFs throughout metazoan evolution. Owing to its low complexity, the COE family constitutes a well-suited paradigm for studying evolution of TF structure and function, including the specific question of protein modularity.

Pioneering analysis of EBF identified three functional domains [1,17]: an amino-terminal, about 210 amino-acid long DBD which is the signature of COE proteins; ii) a Helix-Helix dimerisation motif made of two tandemly arranged α-helical repeats showing limited sequence similarity to the HLH motif described in basic helix-loop-helix (b-HLH) proteins; iii) a transcription-activating domain without marked specific signature. The presence of an Ig-like/Plexin/Transcription Factor (IPT) domain between the DBD and HLH domains was also noticed but the function of this domain remains unknown [18,19]. Comparison between Col and EBF showed that the DBD, IPT and HLH domains have been particularly well conserved during evolution. However, one of the tandemly arranged α-helices noted in EBF/Olf-1 was missing. This, and further examination of the Col and EBF primary sequences led us to postulate the existence, in all COE proteins, of an HLH motif distinct from and partly overlapping the motif initially identified in EBF and Olf-1 [2,11]. This motif is designated below as H1LH2 while the vertebrate-specific motif is designated as H2d-H2a, H2d and H2a (d for duplicated, a for ancestral) corresponding to the duplication of the single H2 helix found in *Drosophila*.

To get more insight into the evolutionary history of *coe *genes, we compared in detail their genomic structure between various metazoan phyla. This comparison shows that the metazoan ancestor COE DBD was built from at least 7 separate exons with no evidence for exon shuffling with other gene families. Detailed analysis of various chordate genomes and ESTs indicated that the H2d duplication has taken place in the vertebrate lineage prior to the two rounds of whole genome duplication characterising the origin of this taxon [20]. It thus provides an example of a novel vertebrate characteristic, which may have been fixed early in the gnathostome lineage. It also revealed that the vertebrate-specific H2 duplication originated from a two-step tandem exon duplication. Careful inspection of the intron phases leads us to put forward an original scenario that involves the selection of a new splice donor site, resulting in the formation of a "cassette" H2d exon. We show here that, alike EBF, Col does bind to DNA as homodimer and that the ancestral H1LH2 motif mediates formation of Col/EBF homodimers and heterodimers. Incorporation of H2d in all four mammalian EBF proteins reveals an interesting example of compulsory counter-selection of alternative splicing following exon duplication.

## Methods

### Search for COE/EBF related sequences in genomic and ESTdatabases

Systematic searches for COE/EBF proteins were conducted in available databases using the BLAST algorithm and mouse COE sequences (Additional file 1) as query. The databases analysed included current versions of the genomes of *Monosiga brevicollis*, *Nematostella vectensis*, *Capitella capitata*, *Lottia gigantae*, *Branchiostoma floridae *[21], of *Strongylocentrotus purpuratus *[22] as well as the Ensembl databases of predicted proteins of *Ciona intestinalis*, *Homo sapiens*, *Mus musculus*, *Monodelphis domestica*, *Ornithorynchus anatinus, Gallus gallus, Xenopus tropicalis *and *Danio rerio *[23]. In *Petromyzon marinus*, the genomic scaffolds containing COE/EBF coding sequences were retrieved from the pre-Ensembl genome version available at [24]. Coding sequences were identified in these scaffolds with GeneWise [25] and assembled using homologous sequences (>95% identity) identified from *Lampetra fluviatilis *ESTs as templates. A survey of ESTs annotated in the databanks for alternative transcript variants included the use of AceView [26]. The sponge *Amphimedon queenslandica *COE sequence was taken from [5].

### Molecular phylogenetic analysis

The alignment of Coe/EBF protein sequences was obtained using MUSCLE [27] and checked by hand under Bioedit [28]. Only full length sequences and unambiguously aligned segments were retained for the phylogenetic analysis (see Additional file 2). Neighbor-Joining (NJ), Maximum likelihood (ML) and bayesian (BI) phylogenetic reconstructions were conducted using the Mega3.1 software, PhyML [29] and MrBayes 3.0 [30]. In each case, we used the JTT model of sequence evolution with invariant+gamma distribution rates. Bootstrap proportions (BP) were calculated by analysis of 1000 replicates for NJ and by the RELL method [31] on the 2000 top-ranking trees for ML analyses. In the BI analysis, four chains were run for 2 million iterations with default heating parameters and sampled every 500 iterations; the first 2000 trees were discarded as burn-in.

### In vitro translation and Electrophoretic Mobility Shift Assays

The pEThBF1 [17], pET15b^His^-mEBF1 (a gift from J. Hagman) and pET17b^His ^Col plasmids and deletions therein were used for in vitro transcription/translation of EBF, EBF*, EBFΔH1, EBFΔH2, Col, Col*, ColΔH1, ColΔH2 and ColΔH2L. To generate internal deletions corresponding to the H1 and H2 helices, we used the four oligonucleotides PCR method [32]. In vitro transcription and translation using rabbit reticulocyte lysate was as described by the manufacturer (kit L1170, Promega). For each protein synthesised, the efficiency of translation was assessed by SDS PAGE of parallel translation reactions performed in the presence of ^35S^methionine. Electrophoretic mobility shift assays (EMSA) were performed in the conditions described by [33], using either a 125 bp DNA fragment containing mb-1 promoter sequences (from -250 to -115) which includes the EBF binding site 5'-AGACTCaaGGGAAT-3' or the PAL probe which contains the palindromic site 5'-ATTCCCaaGGGAAT-3' [1,33] and data not shown. Competition experiments were performed using a 100× molar excess of 30 bp oligonucleotides containing either the wild type 5' -CTAGAGAGAGACTCAA**GG**GAATTGTGGCCAGCCC- 3' or mutated CTAGAGAGAGACTCAA**CC**GAATTGTGGCCAGCCC- 3' mb-1 recognition site, as described in [17].

### Fly strains

The *P [col5cDNA]; col*^1 ^strain designated in Fig.S4 as *col*^1 ^and *UAS-col *strains have been described in [34]. The *P [col5cDNA] *transgene rescues the embryonic lethality but not the wing defects of *col*^1 ^mutants. The *UAS-Mm ebf *and *UAS-Mm ebf2 *constructs were made by cloning the entire *ebf/ebf2 *open reading frame in the pUAST vector. Three independent lines were used for ectopic expression assays. All other stocks were obtained from the Bloomington Stock Center and described in Flybase [35].

## Results

### The coe gene family

**Figure 1:**
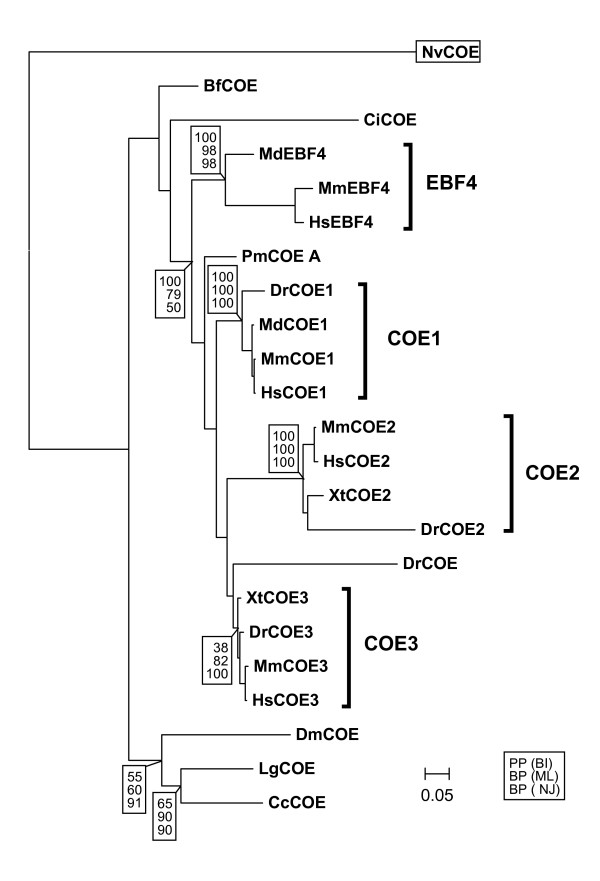
**Bayesian tree showing the phylogenetic relationships between metazoan COE/EBF proteins**. Squared numbers indicate the statistical supports supporting the corresponding nodes : top line, posterior probabilities (x100) in BI, second and third lines, bootstrap proportion (%) obtained in ML and NJ analyses respectively. The statistical supports are only shown for the monophyletic groups obtained in all three reconstruction methods used (protostomes, lophotrochozoans, vertebrates, COE1, COE2, COE3 and EBF4). Those obtained inside the four vertebrate monophyletic groups were omitted for sake of clarity. The tree was rooted using the *N. vectensis *sequence (squared). Species abbreviations : Mm, *Mus musculus *(mouse); Hs, *Homo sapiens *; Md, *Monodelphis domestica *(opossum); Xt, *Xenopus tropicalis *; Dr, *Danio rerio *(zebra fish) ; Pm, *Petromyzon marinus *; Bf, *Branchiostoma floridae *; Dm, *Drosophila melanogaster *; Lg, *Lottia gigantae *(mollusc) ; Cc, *Capitela capitata *(annelid) ; Nv, *Nematostella vectensis *(sea anemone). The scale bar represents the number of substitutions per amino-acid along each branch. Accession numbers are indicated in table S1.

The recent identification of *coe *sequences in cnidarians and poriferans [4,5], together with the absence of evidence for *coe *genes outside metazoans suggests that COE proteins have appeared with this taxon. In line with this conclusion, no COE-related sequence could be identified in the genome of the choanoflagellate *Monosiga brevicollis*, while COE sequences have been reported in the sponge *Amphimedon queenslandica *and the sea anemone *Nematostella vectensis *[4,5]. In order to obtain an exhaustive characterisation of COE proteins and their relationships in metazoans, we first updated the phylogenetic analysis available for this family [4], taking advantage of the wide range of genomes now available (Fig. 1 and see Additional file 2). Systematic searches for *coe/ebf *related sequences were carried out in the genome of a diploblast, two ecdysozoans (fly *Drosophila melanogaster *and nematode *Caenorhabditis elegans*), two lophochotrozoans (annelid *Capitella capitata *and mollusc *Lottia gigantae*), an echinoderm (sea urchin Strongylocentrotus purpuratus), the cephalochordate *Branchiostoma floridae*, the ascidian *Ciona intestinalis *and eight vertebrates, including the lamprey *Petromyzon marinus*, the platypus *Ornithorhynchus anatinus *and the oppossum *Monodelphis domestica *in addition to the mouse, human, chick, xenopus (*X. tropicalis*) and zebrafish (see Additional file 1 for accession numbers and nomenclature of each gene). A single *coe *gene was found in all metazoans studied, except in vertebrates, with evidence for three genes in *Xenopus*, and a fourth one in the four mammals studied as previously reported in the mouse and human [4], but also in the zebrafish. In *P. marinus*, two distinct clusters, spanning the 5' part of the coding region and including the HLH region were reconstructed from the genome, thus pointing to the presence of at least two *coe *genes in lampreys. The phylogenetic analysis was conducted using NJ, ML and bayesian algorithms, excluding highly divergent sequences as well as heavily truncated ones but retaining representatives of ecdysozoans, lophotrochozoans and of the major chordate taxa (see Additional file 2). In the resulting trees (Fig. 1), protostome, as well as lophotrochozoan coe sequences were found clustered in monophyletic groups in NJ, ML and BI, albeit with low statistical supports except in NJ (BP= 91 and 90 respectively). Similarly, the monophyly of the vertebrate sequences retained in the reconstruction was retrieved whatever the algorithm used, with moderate to good statistical supports in BI and ML (respectively PP = 1 and BP = 79; but BP = 50 in NJ), thus supporting and extending the results obtained by Pang et al., 2004. Inside this group, all three reconstruction methods also confirmed the previously reported presence of four additional monophyletic groups. Three of them contain at least one zebrafish or one *Xenopus *sequence in addition to one mammalian sequence (either COE1, COE2, or COE3). These groups were named accordingly COE1, COE2, both strongly supported (PP = 1 in BI, BP = 100 in NJ and ML), and COE3, less well supported in BI (PP = 0.38, but BP = 82 in ML and 100 in NJ). The fourth group (PP = 1 in BI, BP = 98 in ML and NJ) only contains mammalian sequences, clustering with mouse EBF4. Together with the identification of COE1, COE2, COE3 and EBF4 partial sequences in the platypus *O. anatinus *(excluded from the reconstruction due to truncations in the available sequences: see Additional file 3), this clearly indicates that the emergence of the EBF4 class has predated the mammalian radiation. The branching order observed for this group, always found as a sister group of all other vertebrate sequences (albeit with poor statistical supports), may be taken as evidence for an ancient origin in the vertebrate lineage, with subsequent losses in actinopterygians, amphibians and archosaurs (the three chick genes appearing respectively related to the COE1, COE2 and COE3 classes, see Additional file 3). However, a reconstruction artefact possibly related to the relatively long branches observed in this group among mammals remains difficult to exclude. Finally, the relative branching orders of the lamprey sequence included in the analyses (termed PmCOE-A) and of one of the zebrafish sequences (termed DrCOE) (Fig. 1) were found to vary depending on the algorithm and could not be resolved. Altogether, the phylogenomic analysis supports the conclusion that all vertebrate *coe *genes included in the reconstruction are derived from a single ancestral *coe *gene, present in the vertebrate lineage prior to the splitting between gnathostomes and cyclostomes. It also confirms the presence of four COE classes in gnathostomes. The emergence of three of them (COE1-3) is likely to have been linked to the two rounds of whole genome duplication that have occurred in the vertebrate lineage prior to the gnathostome radiation, while the origin of the fourth one (EBF4), which only contains mammalian sequences, is less clear. Finally, the chronology of the corresponding gene duplications relative to the cyclostome-gnathostome divergence, as well as the relationships of the lamprey genes with the four gnathostome classes, remained unresolved. Even though the number of genes identified in the lamprey and mammals (2 versus 4) is suggestive of the occurrence of a first round of duplication prior to the cyclostome-gnathostome splitting and a second one after their divergence, the phylogeny does not allow firm conclusions on this point.

### The metazoan ancestor COE DBD was built from multiple "unique" exons

**Figure 2:**
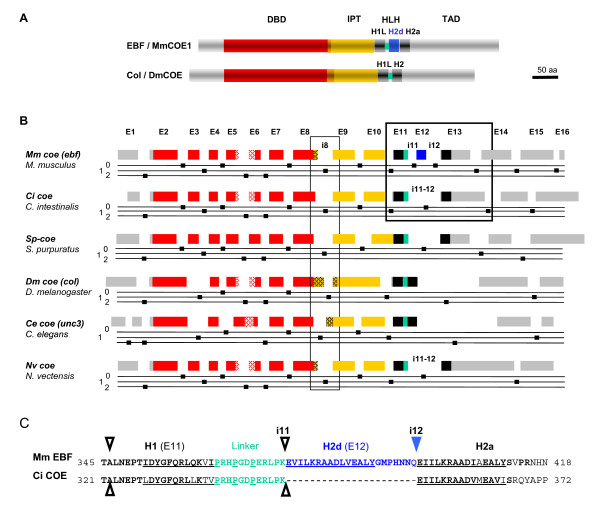
**Compared structure of the COE proteins and *coe *genes in representative metazoan phyla**. (A) Schematic representation and alignment of mouse EBF/COE1 and *Drosophila *Col. The regions corresponding to the DNA Binding Domain (DBD) and the IPT domain are shown in red and yellow, respectively. The ancestral Helix-Loop-Helix (HLH) motif is represented by two separate black boxes (helices H1 and H2a) and the H1-H2 linker in green. The duplicated helix (H2d) specific to the vertebrate proteins is indicated by a blue box and the C-terminal transactivation domain is in grey. (B) Evolutionary conservation of the *coe *splice structure. The positions of introns are indicated relative to the different COE protein domains, using the same color code as in (A). Each exon is numbered using the genomic structure of human *ebf1 *as a reference. The class (0, 1 or 2) of each intron is indicated by a small dot on the corresponding line below each gene. The HCCC zinc finger in the DBD is red dotted. The short linker of variable length and sequence separating the DBD and IPT domains is yellow dotted. The genomic organisations of the DBD/IPT linker region in all COE proteins and the HLH domain in EBF and Ci-COE are surrounded. (C) Sequence alignment between the HLH motifs of EBF and Ci-COE, using the same colour code than in (A). The similarity region of the H1, H2d and H2a α-helical repeats with the HLH repeat of b-HLH proteins is underlined. The duplicated segment in vertebrate proteins extends a few amino acids beyond the predicted α-helical region. The open arrowheads indicate the positions of ancestral introns, with their number indicated. The blue downwards pointing arrowhead indicates the position of the novel intron of vertebrate *ebf *genes.

In vitro functional dissection of EBF, when EBF was a pioneer protein, delineated the COE DBD which turned out to be unrelated to other, previously characterised DBDs, except for the presence of a zinc coordination motif [1,17]. Sequence conservation between EBF1 and *Drosophila *Col then showed that this DBD constituted the molecular trademark of a new family of transcription factors designated as COE proteins [2,11]. Sequence alignments between representative members of different phyla highlight the high degree of evolutionary conservation of each of the three COE-specific domains, the DBD, IPT and atypical HLH domains as well as scattered blocks of sequence similarity in the carboxy-terminal transactivator domain (TAD) (Fig. 2A and see Additional file 2). Fig. 2b shows a diagrammatic comparison of the exon-intron structure of *coe *genes between representatives of deuterostomes, including the chordate (Craniata) *Mus musculus*, the urochordate ascidian *C. intestinalis *and the echinoderm *Stongylocentrus purpuratus *and representatives of protostomes, including the insect *D. melanogaster *and the nematode *Caenorhabditis elegans *and the cnidarian *N. vectensis*. An immediate outcome from this comparison was the remarkable conservation in number and positions of introns, independent of the overall size of the *coe *trancription units which ranges from around 10 kb in *C. elegans *[10] to around 400 kb in mouse *ebf1 *[23]. This showed that the ancestor *coe *gene was built from a complex set of multiple exons, and that this highly fragmented structure was maintained throughout metazoan evolution. Functional domains in proteins are, on a large scale, associated with protein coding exons in the genome [36]. It therefore came to us as a surprise to find that the COE DBD was constructed from at least seven separate exons, since introns are generally thought not have functions [37] although more recent reports tend to suggest that intron accumulation in conserved genes might be an adaptive process [38]. Since each of the 6 introns interrupting the DBD is found at the same position and in the same phase in deuterostomes, protostomes and cnidarians, we conclude that this splice structure is ancestral with the variation of exon number observed, for example in *Drosophila melanogaster *and *C. elegans *reflecting secondary lineage-specific loss of introns (Fig. 2B and not shown; [39,40]. Similarly, introns interrupting the IPT and HLH domains are also lost in *D. melanogaster *(Fig. 2b) Contrasting with the conservation of length and primary sequence of the DBD and the IPT+HLH domains, significant primary sequence variation between different phyla is observed at the junction between the DBD and IPT domains (see Additional file 2). This correlates with a variable position of the intron separating these two domains (intron i8 in *ebf*, Fig. 2B) within a "linker" region whose sequence and length is itself variable (see Additional file 3). Further sequence variation in this region is conferred by the use of two different E8 splice donor sites, resulting in EBF isoforms differing by the inclusion or not of a 8 to 10 amino acids, the possible functional consequences of which remains to be addressed. Domain exchange and/or accretion between proteins resulting from exon shuffling is believed to be one of the driving forces behind protein evolution. During this process, symmetrical exons,*i.e*. exons flanked by same-phase introns, can be either deleted, duplicated or inserted, without disrupting the downstream protein reading frame [41]. Since, except for exon E6, all the exons contributing the COE DBD are asymmetrical (Fig. 2B), this domain could not be constructed from accretion of subdomains present in other bilaterian proteins through exon shuffling. Consistent with this conclusion, systematic blast-search analyses with individual DBD exons failed to retrieve proteins other than COE proteins from databanks. A comprehensive theory to explain intron abundance and high level of position conservation among species is still missing [42]. How the COE-specific DBD structure was put together in first place remains therefore a fascinating question.

### The modified HLH motif of EBF proteins is a vertebrate innovation

A noticeable difference between EBF and *Drosophila *Col is the specific duplication of a short α-helical region in EBF (H2d-H2a tandem repetition, Fig. 2A,C). Because of remote sequence similarity, this H2 tandem repetition was originally proposed to constitute a dimerisation similar to that present in b-HLH proteins found in fungi, plants and metazoans [1,3,43]. The absence of H2d in *Drosophila *Col led us, however, to propose the existence of an alternative Helix-Linker-Helix (H1LH2) domain [2,11]. Sequence comparison of a wide range of metazoans shows that the H1LH2 motif is an ancestral character (see Additional file 2). The predicted primary sequence of COE proteins in cephalochordates and urochordates, which are considered as the closest living relatives of the vertebrate ancestor [12,44,45], suggested that the H2d-H2a duplication was a vertebrate-specific feature. To confirm a conclusion mostly based on ESTs analysis, we retrieved the intronic sequences comprised between the H1 and H2a coding exons in representatives of the major chordate groups outside vertebrates, *C. intestinalis*, *B. floridae *and *S. purpuratus *and verified the absence of H2d-related coding sequence (see Additional file 3). In contrast, all the gnathostome sequences retrieved from the genomes analysed, including not only the four COE1-3 and EBF4 classes but also the unassigned zebrafish DrCOE sequence, exhibit the H2d addition, strongly suggesting that the H2a-H2d duplication predated the gnathostome radiation (see Additional file 2). In the lamprey *P. marinus*, we found no evidence for the presence of H2d in the *Pmcoe-A *locus, but a definitive conclusion could not be obtained in this case due to sequence gaps between the H1 and H2a coding regions in the current genome version. In contrast, the presence of H2d could be unambiguously recognised, and at the expected position, in the deduced PmCOE-B amino acid sequence (see Additional file 3). Preliminary EST analyses of a closely related lamprey species, *Lampetra fluviatilis*, confirmed the presence of H2d in transcripts of the orthologous *Lfcoe-B *gene but also highlighted the presence of alternatively spliced forms, devoid of the duplicated H2 sequence (see Additional file 4). The presence of H2d in lamprey LCOE-B may thus be subject to alternative splicing, while no indication for a similar process has been obtained thus far in gnathostomes. Taken together, these data indicate that the H2 duplication occurred early in the vertebrate lineage, prior to splitting between gnathostomes and cyclostomes, in a single copy ancestral gene from which all gnathostome and at least the lamprey *coe-A *genes are derived. They also suggest that this additional protein domain may have been fixed early in the gnathostome lineage.

### The COE-HLH dimerisation motif revisited

**Figure 3:**
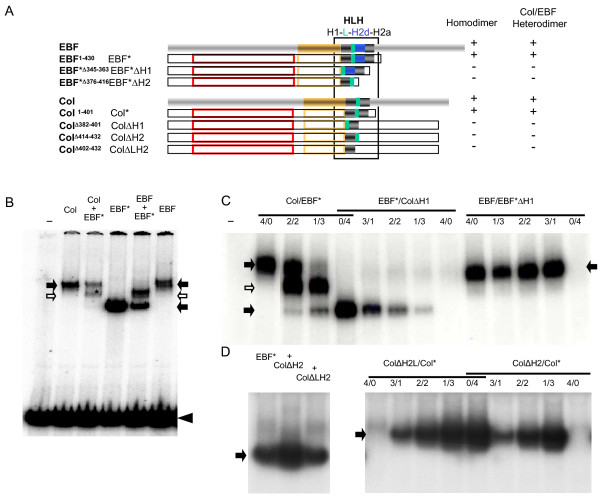
**The HLH motif mediating the formation of COE dimers**. (A) Diagrammatic representation of full-length mouse EBF/COE1 and *Drosophila *Col and various carboxy-terminal or/and internal deletions used for Electrophoretic mobility shift assays EMSA (see also Additional file 4). The colour code is as in Fig.1. The deleted helix motif is preceded by Δ in the second column. The homodimer and heterodimer (EBF/Col) columns summarize the data from EMSA;. (B,C,D) EMSA with the ^32^P labelled *mb-1 *probe and variants of Col and EBF as indicated above each lane. Homodimers are indicated by black arrows and heterodimers by open arrows, respectively, with the presence of a truncated form of either Col or EBF indicated by*. The free probe is marked by an arrowhead (only shown in B). (B) Col or EBF and EBF* are able to form homo and heterodimers. (C) The H1 α-helix is required for either Col or EBF* to form dimers. (D) The H2 α-helix is also required for Col to form either homodimers or heterodimers with EBF*, left and right lanes, respectively. Two different deletions were tested (see A).

EBF/Olf-1 was initially isolated as a nuclear factor recognising functionally important cis-regulatory DNA sequences in the promoter of *mb-1*, an early B-lymphocyte specific gene and olfactory marker protein genes [46,47]. Further characterisation showed that EBF/Olf-1 recognises variations on the palindromic sequence TTCCCNNGGGAAT and binds DNA as a homodimer [1,3,33]. It was thus proposed that homodimer formation was mediated by the H2d-H2a α-helical repetition [1,3,17] (see Fig. 2A). The absence of H2d in COE proteins outside vertebrates (Fig. 2C) raised, however, the question of whether all COE proteins could bind DNA as dimers and, if so, which motif was involved in dimer formation. We therefore proposed that the H1LH2a motif that is found in all COE proteins was playing this role. To experimentally address this question, we compared the dimerization properties of *Drosophila *Col and EBF and modified versions of these two proteins (Fig. 3A and see Additional file 5), using gel-shift assays with the vertebrate *mb-1 *promoter DNA. We first found that Col forms complexes with *mb-1 *DNA that migrate at the same position than EBF/*mb-1 *complexes, without evidence of fast migrating complexes which would correspond to monomers (Fig. 3B). We could therefore conclude that, similar to EBF, Col binds to DNA as homodimer. Truncated forms of EBF and Col, that lack the transactivation domain (designated below as EBF* and Col*, respectively, Fig. 3A) form complexes of higher mobility than the full-length proteins ([1], Fig. 3B,D). We took advantage of this higher mobility to assay Col ability to form heterodimers with EBF, using a mixture of full length Col or EBF and EBF*. The formation of three types of DNA/protein complexes (Fig. 3B,C) indicated that Col is able to form heterodimers with EBF. Interestingly, Col/EBF heterodimer formation was favoured over homodimer formation (Fig. 3B,C and data not shown). Since binding to mb-1 DNA is an indirect assay for dimer formation, this observation which could indicate either favoured heterodimerization or higher DNA binding affinity of heterodimers needs to be further investigated. Above all, these data indicated that H2d is not required for dimerisation of COE proteins. Conversely, a truncated EBF-4 protein containing H2d but lacking H2a (OE-4S) was reported to bind to DNA as homodimer [7]. Together, these data allow to conclude that the presence of a single copy of H2 is sufficient for binding of COE proteins to bind DNA as dimers. We then tested the specific requirement both for H1 and H2 (H2a or H2a and H2d), by precisely removing either helical domain in Col or EBF* (ColΔH1/ColΔH2a and EBF*ΔH1/EBF*ΔH2 proteins, respectively Fig. 3A). Neither deleted protein form was able to bind to DNA, when co-expressed with either EBF* or Col* (Fig. 3C,D and not shown). This led us to conclude that the presence of both H1 and at least one H2 are essential for COE dimer formation and that the ancestral H1-L-H2a mediate dimerisation of COE proteins.

### A two-step evolutionary scenario for inclusion of H2d in vertebrate EBF

**Figure 4:**
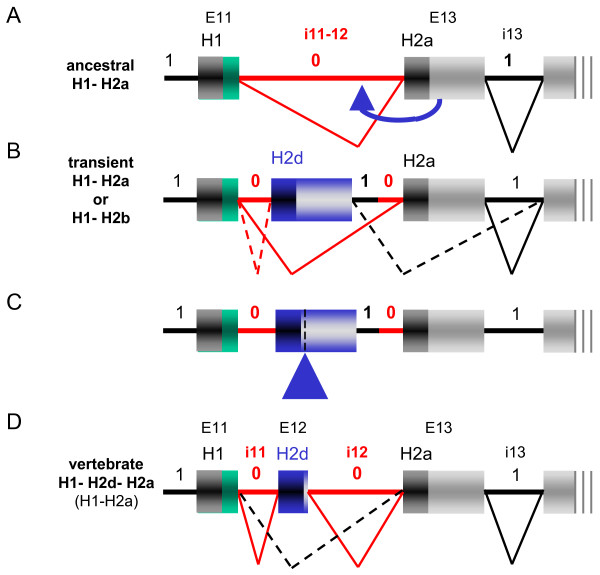
**A two-step scenario for the H2 duplication in vertebrate COE proteins**. (A) Exon-intron structure of the H1-H2a HLH motif in the *coe *ancestor gene; the phase of introns is indicated, with phase 0 introns in red and phase 1 introns in black. (B) The first step in H2 duplication was a duplication of the exon encoding H2a. Because of the splice phase rule, this could only result in alternative splicing out either the ancestral (H2a, dotted angled line) or the newly created exon (H2d, solid angled line). (C) The second step was the activation of a cryptic 3' splice donor site, downstream of the H2d coding region (blue arrowhead). This resulted in same phase i11 and i12 introns and the incorporation of H2d into COE proteins (D). Note that H2d exon-skipping can theoretically occur (dotted angled line).

All gnathostome *coe/ebf *cDNAs analysed to date include H2d. To investigate the possible mechanisms behind this inclusion, we compared in detail the genomic structure of the HLH region between gnathostomes and their closest relatives, the urochordates [45], for which genomic sequences are available. Each of the 3 α-helical repeats present in human EBF (H1, H2d and H2a) is encoded by a separate exon, (exons E11, E12 and E13, separated by introns i11 and i12, respectively, Fig. 2B,C). An intron also found between H1 and H2a in *C. intestinalis Ci-coe*, designated i11-12 must have predated the separation between urochordates and vertebrates. The presence of this intron in other deuterostomes, the cephalochordate *B. floridae *and the echinoderm *Strongylocentrus purpuratus *but also the cnidarian *N. vectensis *(Fig. 2B) confirmed its ancestral character (Fig. 4A). Both i11, i12 and i11/12 are phase 0 introns, which could indicate a simple scenario whereby a tandem duplication of the exon encoding ancestral H2a would be at the origin of vertebrate H2d. Such a straightforward scenario is not compatible, however, with the position and phase of the 3'-next intron (i13) which, both in *C. intestinalis *and mammals, is neither situated immediately downstream of the H2 coding sequence nor a phase 0 but a phase 1 intron (Fig. 2B and 4A,B). Since only symmetrical exons can be inserted into introns, of the same phase, without disrupting the downstream reading frame, this observation rules out a simple exon E12 duplication and implies a secondary event. We therefore propose the following two-steps model: First, a duplication of exon E13 (Fig. 4B) led to a situation where either one of the ancestral or duplicated exon, but not both, could be incorporated in the mature mRNA, a classical case of mandatory alternative splicing [48,49]. Second, selection of a new splice donor site, a few nucleotides downstream of the H2d coding region, restored a phase 0 intron (Fig. 4C), allowing both H2a and H2d to be inserted into the reading frame (Fig. 4D). Other possible scenarii were envisaged, using different intronic recombination events, but none appeared to be more parsimonious than the two-step scenario that we put forward here. Of the two vertebrate H2, the C-terminal is the more closely related to the single H2 of invertebrates (Fig. 2D and S2), indicating that the duplicated helix has started to diverge. The "cassette" H2d exon can theoretically be inserted or removed from the transcript without affecting the rest of the protein. Removal of the H2d exon from the mature *ebf *mRNAs through exon-skipping (Fig. 4D, dashed line) would restore an invertebrate-like protein. However, we could not find evidence for H2d exon skipping in gnathostome COE proteins, either by surveying ESTs annotated in the databanks (AceView; [26]) or by PCR amplification of this coding region in mouse *coe1*, *coe2 *and *coe4*, using specifically designed primers with mRNA from several different tissues (data not shown). Therefore we conclude that in gnathostomes, the prevalent form of COE proteins results from the compulsory inclusion of H2d. This inclusion therefore represents an interesting case of counter-selection of exon-skipping, a mechanism widely used in vertebrates to amplify the register of protein products and their differential expression during development [49,50].

## Discussion

The COE family of transcription factors was first defined by the sequence similarity between rodent EBF/Olf-1 and *Drosophila *Col [1-3]. Cloning of a *coe *cDNA from the cnidarian *N. vectensis *and identification of *coe *sequences in another cnidarian, *hydra magnipapillata *and a poriferan, the sponge *Amphimedon queenslandica *[4,5] strengthened the conclusion that *coe *genes are metazoan-specific genes. Our systematic blast-search for *coe *orthologs in DNA sequence databanks confirmed that *coe *genes are metazoan genes present at a single copy per genome, except for vertebrates. It further showed a remarkable degree of conservation of the *coe *genomic structure throughout metazoan evolution, except for one exon duplication in the vertebrate lineage.

### The scattered structure of coe genes

All introns found in the *cnidaria N. vectensis Nvcoe *gene are also found, at the same position, in deuterostomes and at least one of the protostomes examined, suggesting that this scattered organisation was already present in the metazoan ancestral *coe *gene. In case of the DBD, which is both specific of COE proteins and conserved to the same degree over its entire length, a split structure into 7 exons was rather unexpected. Moreover, we could not find evidence for exon shuffling with other gene families, consistent with the conserved asymmetric intron phases, but leaving intact the question of the genomic building up of this unique DNA binding domain. The HCCC zinc finger structure proposed to be an essential feature of the EBF DNA-binding domain [17] is itself encoded by two exons, already in the last common cnidarian/bilaterian ancestor (E5 and E6, Fig. 2B), suggesting a bipartite origin. Since exon E6 is symmetrical, it can possibly be subject to regulated exon-skipping, allowing for the production of different protein isoforms, with putatively different functions. Whereas there is some preliminary evidence for it, as a subclass of human EBF1 cDNAs may differ from the main class by the loss of exon E6 (AceView; [26] the i5 intron has been lost in some protostomes, such as *Drosophila melanogaster *(Fig. 2B). Systematic genome sequencing programs should soon give access to the *coe *gene structure in many additional phyla, including sister clades of bilateriae. It offers the exciting prospect of deeper insight into the evolutionary roots of the *coe *gene family and their scattered genomic organisation.

### The ancestral COE HLH motif revisited

Sequence similarity of the ancestral COE H1LH2 motif with the HLH motif of basic-HLH proteins [43] has led to classify COE proteins as one distant subgroup in this superfamily of proteins, despite displaying distinctive DBD and additional protein domains [5,51]. In vitro DNA binding assays show that the H1LH2 motif is required for binding of COE proteins to DNA as dimers. This conclusion differs from the initial report that the EBF dimerisation motif was H2d-H2a, a conclusion supported by the analysis of two different deletions in EBF. Indeed, an internal deletion of EBF removing amino acids 296 to 367 (EBFΔ^296–367^), namely H1 and part of the IPT domain, was reported to lower but not prevent dimer formation [1]. Since we found that removal of H1 alone in either EBF or Col abolished dimer formation, one possibility is that the presence of the IPT domain interferes with the ability of the H2d-H2a repeat to mediate homophilic interactions. In support of this possibility, the H2d-H2a repeat, when taken out of its normal context, is able to promote formation of dimers, as shown by using a truncated nuclear hormone receptor lacking its own dimerization domain [17]). The high degree of sequence conservation of the COE IPT domain (see Additional file 3) suggests that this domain is subject to very stringent structural and functional constraints. Together, our results from DNA-binding assays and those reported by [1], further suggest that the positioning of the IPT and HLH domains in relation to one another is a critical aspect of COE dimer formation. Hagman et al; 1995 [17] also reported that a modified EBF protein lacking amino acids 370 to 383 (EBFΔ^370–383^), i.e., part of H2d, leaving intact H2a (see Additional file 5), showed a drastically reduced level of binding to *mb1 *DNA, suggesting that H2d was essential for forming EBF homodimers. Yet, the 370 to 383 a.a. deletion does not only remove part of H2d but also part of the linker separating H1 and H2 (see Additional file 5). Our data suggest that it is removal of this linker rather than H2d itself which prevents EBF dimer formation. The conservation of sequence and genomic structure of this Proline-rich linker throughout metazoan evolution (see Additional files 2 and 3) supports a key role in positioning H1 and H2 relative to each other and contribution to the DNA-binding specificity of COE dimers. While efficient *in vitro *binding to DNA of either Col dimers, Col/EBF heterodimers or dimers of EBF isoforms lacking either H2a (Fig. 3) or H2d [7] indicates that the H2 duplication is not essential for EBF dimer formation, inclusion of a duplicated helix2 raises the interesting possibility that it could result in an increased partnership flexibility and functional versatility of the vertebrate COE proteins. The observation that Col/EBF heterodimers more efficiently form and/or bind to DNA, at least in vitro, raises the speculative hypothesis that it could have been the initial force behind the selection of H2 exon inclusion.

### Counter-selection of alternative splicing

Together, the compared structures of vertebrate and urochordate *coe *genes between echinoderms, cephalochordates, urochordates and a wide range of vertebrates, including cyclostomes suggest that the duplication of H2 occurred in the vertebrate ancestor and resulted from an exon-duplication event. This is the only major change in the modular structure of COE proteins that appears to have been fixed throughout metazoan evolution. Exon duplication is one widely used mechanism for adding a coding region within an existing gene. Alternative splicing of duplicated exons has been postulated to favor protein diversification, since each exon can, in principle, evolve independently of the other [48,49]. Recent genomic studies have suggested that 40–60% of human genes are alternatively spliced and comparative analysis of close to 10,000 orthologous genes in human and mouse has shown that alternative splicing is frequently associated with recent exon creation and/or loss [52]. However, other studies suggest that the contribution of gene duplication, followed by sequence divergence and alternative splicing to the diversification of the protein repertoire could be substantially different [53]. In the case of the vertebrate *coe *genes, alternative splicing was not selected by evolution following exon H2d duplication, since both H2 repeats are incorporated in the EBF proteins. Taking into account the splice frame rules, we put forward here an original two-step model to account for the inclusion of H2d in vertebrate COE proteins (Fig. 4). The first step in our model is a classical tandem duplication of an "ancestral" H2a coding exon. However, this exon was probably not symmetrical (see Fig. 2B) and, due the splice frame rule, only the ancestral or the duplicated exon could be incorporated in the coding transcript without disrupting the open reading frame, a classical case of mandatory alternative splicing [48,49]. We believe that inclusion of the duplicated exon occured via the activation of a phase 0 splice donor site, 3' to H2 in the duplicated exon This allowed the incorporating of H2d, while preserving the open reading frame (see Fig. 4D). To our knowledge such a two-step selection of a cassette exon has not yet been invoked for other proteins.

While our data underline the conservation of *coe *protein structure throughout evolution, the molecular mechanisms underlying the cell-context dependence of COE regulatory targets remains unknown. For example, mouse EBF/COE1 or EBF2/COE2 can substitute for Col activity in UAS-Gal4 transgenic assays [54], using as a paradigm Col function in patterning of the wing [34], indicating that Col and EBF are able to regulate similar set of genes in a tissue-dependent manner (see Additional file 6). So far, little insight was obtained from systematic searches for EBF or Col directly protein interactors [55,56]. This remains a pre-eminent question in view of the evolutionary diversification of the biological functions of COE proteins revealed by mutant analyses in both mouse, *C. elegans *and *Drosophila *[57-60]. Within this context, more extensive analysis of genomic structure, expression and function of COE proteins in other phyla could be of primary interest.

## Conclusion

Our systematic blast-search for *coe *(*collier/olf-1/ebf*) orthologs in DNA sequence databanks confirmed that *coe *genes are metazoan genes present at a single copy per genome, except for vertebrates. It further showed a remarkable degree of conservation of the *coe *genomic structure throughout metazoan evolution, except for one exon duplication in the vertebrate lineage, leading to a modified dimerisation domain of structure H1lH2dH2a in vertebrates and HLH2a in all other metazoans. Taking into account the splice frame rules, we put forward here an original two-step duplication model to account for H2d inclusion in vertebrate COE proteins The vertebrate gene configuration is such that it remains possible to remove H2d through alternative splicing, through exon-skipping. However, the presence of both H2d and H2a in all gnathostome *coe*/*ebf *transcripts characterised to date both indicates that, in this case, exon-skipping is highly counter-selected. While *in vitro *experiments indicate that the H2 duplication is not essential for binding of COE proteins to DNA as dimers, it raises the interesting possibility that it could result in an increased partnership flexibility and functional versatility of the vertebrate COE proteins.

## Authors' contributions

VD and SeM carried out the experimental work, SeM, J–LP and SyM carried our the phylogenic analyses, SeM, MC and AV contributed the conceptual framework and SyM and AV wrote the manuscript. All authors read and approved the final manuscript.

## Supplementary Material

Additional file 1 **Supplementary Table.** Identification of the metazoan COE sequences used in this study. Click here for file

Additional file 2 Sequence alignments used in the phylogenetic analysis of metazoan COE proteins. Click here for file

Additional file 3 Alignment of the DBD, IPT and HLH domains of the COE proteins. Click here for file

Additional file 4 Structure of *P. marinus *and *L. fluviatilis *COE-B transcripts at the level of the H2d coding region. Click here for file

Additional file 5 Diagrammatic alignment of EBF and Col protein amino-acid sequences. The sequence alignment provided shows the position of the H1 and H2 deletions introduced in modified versions of EBF and Col used in DNA-binding assays (Fig. 3) and EBF internal deletion described in Hagman et al., 1995 [17]. Click here for file

Additional file 6 EBF can substitute for Col in patterning of the *Drosophila *wing. This figure compares the phenotypes of adult Drosophila wings subject to targeted expression of either mouse EBF1 or EBF2 or *Drosophila *Col. Click here for file
