# Supplementary material for: The metazoan history of the COE transcription factors. Selection of a variant HLH motif by mandatory inclusion of a duplicated exon in vertebrates
Source: BMC Evol Biol. 2008 May 2;8:131. doi: 10.1186/1471-2148-8-131 (PMC2394523; doi:10.1186/1471-2148-8-131)
Supplement: Additional file 1 — Supplementary Table. Identification of the metazoan COE sequences used in this study. [file 1471-2148-8-131-S1.pdf]

```
                                                                                                               DBD
GNATHOSTOMES
COE1
Mm COE1     ---------- ---------- -M-FGIQES- -----I-QRS G-SSMKEE-P L-GS---GMN --AVR-TWMQ GAGVLDANTA AQSGVGLARA HFEKQPPSNL RKSNFFHFVL
Hs COE1     ---------- ---------- -M-FGIQES- -----I-QRS G-SSMKEE-P L-GS---GMN --AVR-TWMQ GAGVLDANTA AQSGVGLARA HFEKQPPSNL RKSNFFHFVL
Md COE1     ---------- ---------- -M-FGIQES- -----I-QRS G-SSMKEE-P L-GS---GMN --AVR-TWMQ GAGVLDANTA AQSGVGLARA HFEKQPPSNL RKSNFFHFVL
Xt COE1     ---------- ---------- ---------- ---------- ---------- ---------- ---------- ---------- ---------- ---------- ----------
Dr COE1     ---------- ---------- -M-FGIQDS- -----I-QRS G-SSMKEE-P I-GA---GMN --AVR-TWMQ GAGVLDANTA AQSGVGLARA HFEKQPPSNL RKSNFFHFVL
COE2
Mm COE2     ---------- ---------- -M-FGIQDT- -----L-GR- G-PALKDK-S L-GA---EMD --SVR-SWVR NVGVVDANVA AQSGVALSRA HFEKQPPSNL RKSNFFHFVL
Hs COE2     ---------- ---------- -M-FGIQDT- -----L-GR- G-PTLKEK-S L-GA---EMD --SVR-SWVR NVGVVDANVA AQSGVALSRA HFEKQPPSNL RKSNFFHFVL
Xt COE2     ---------- ---------- -M-FGVQDT- -----L-ARS G-TALRDK-A L-GV---GMD --PVR-SWVR NVGVVDAKVA AQSGVAVSRA HFEKQPPSNL RKSNFFHFVL
Dr COE2     ---------- ---------- -M-FESQDH- -----S-IRT I-TELKVR-T F-EE---EMD --PVR-SWVR NVGVIDANIA AQSGVALSRA HFEKQPPSNL RKSNFFHFVL
COE3
Mm COE3     ---------- ---------- -M-FGIQEN- -----I-PRG G-TTMKEE-P L-GS---GMN --PVR-SWMH TAGVVDANTA AQSGVGLARA HFEKQPPSNL RKSNFFHFVL
Hs COE3     ---------- ---------- -M-FGIQEN- -----I-PRG G-TTMKEE-P L-GS---GMN --PVR-SWMH TAGVVDANTA AQSGVGLARA HFEKQPPSNL RKSNFFHFVL
Xt COE3     ---------- ---------- -M-FGIQEN- -----I-PRG G-TTMKEE-P L-GG---GMN --PVR-SWMH TAGVVDANTA AQSGVGLARA HFEKQPPSNL RKSNFFHFVL
Dr COE3     ---------- ---------- -M-FGIQEN- -----I-PRG G-TTMKEE-P L-GS---GMN --PVR-SWMH TAGVVDAHTA AQSGVGLARA HYEKQPPSNL RKSNFFHFVL
EBF4
Mm EBF4      ---------- ---------- -M-FPAQDA- -----L-PRG G-LHLKEE-P LLPS---SLG --SVR-SWMQ SAGILDSNTA AQSGVGLARA HFEKQPPSNL RKSNFFHFVL
Hs EBF4      ---------- ---------- ------MDA- -----L-PRS G-LNLKEE-P LLPA---GLG --SVR-SWMQ GAGILDASTA AQSGVGLARA HFEKQPPSNL RKSNFFHFVL
Md EBF4      ---------- ---------- -M-FSMQDS- -----L-PRG G-LSLKEE-P L-PA---GLG --SVR-SWMQ GTGILDATTA AQSGVGLARA HFEKQPPSNL RKSNFFHFVL
Unassigned
DrCOE       ---------- ---------- ---------- ---------- ----MKQE-V R-GS---SAG --SAV-SWLH THSSL--ST- -QRDVGLARA HFEKQPPANL RKSNFFHFVL
LAMPREYS
PmCOE-A     ---------- ---------- -M-FGIQES- -----L-PRG G-SGLKEE-P L-GG---GMP --TVR-SWMH S-GVVDASTA AQSGIALARA HFEKQPPSNL RKSNFFHFVL
NON-VERTEBRATES
Ci COE      ---------- ---------- ---------- ---------- ---------- ---------- ---------- ---------- ----VGLHRA HFEKQPPNNL RKSNFFHFVL
Bf COE      ---------- ---------- ------MDT- -----L-PRG T-TTLKEE-P L-GS---QIG --SVR-NWMQ P-SIVDQT-A TSGSVGLARA HFEKQPPSNL RKSNFFHFVI
Lg COE      ---------- ---------- -M-FVT---- -----D-QET T-SPMKEE-S L-SS---QLS --AVR-NWMQ PT-VLDQN-- --GSVGLSRA HFEKQPPSNL RKSNFFHFVL
Cc COE      ---------- ---------- -M-FSLQDS- -----LQPRG PLSGMKEE-P L-GAPQAPLG --TVR-NWMQ GPSAVDQA-A ATASVGLGRA HYEKQPPSNL RKSNFFHFVL
Dm COE      MEWGRKLYPS AVSGPRSAGG LM-FGLPPTA AVDMNQ-PRG PMTSLKEE-P L-GS------ ------RWAM QP-VVDQ--- --SNLGIGRA HFEKQPPSNL RKSNFFHFVI
Nv COE      ---------- ---------- -MSFDMQEQ- -----D---- E-NSMKNDRP V-V------- ------GCLQ HR-VLEPQVT STASGLLARC HFEKQPPANL RKSNFFHFVL

                                                 DBD
GNATHOSTOMES
COE1
Mm COE1     ALYDRQGQPV EIERTAFVGF VEKEK----E ANSEKTNNGI HYRLQLLYSN GIRTEQDFYV RLIDSMTKQA IVYEGQDKNP EMCRVLLT-- -HEIMCSRCC DKKSCGNRNE
Hs COE1     ALYDRQGQPV EIERTAFVGF VEKEK----E ANSEKTNNGI HYRLQLLYSN GIRTEQDFYV RLIDSMTKQA IVYEGQDKNP EMCRVLLT-- -HEIMCSRCC DKKSCGNRNE
Md COE1     ALYDRQGQPV EIERTAFVGF VEKEK----E ANSEKTNNGI HYRLQLLYSN GIRTEQDFYV RLIDSMTKQA IVYEGQDKNP EMCRVLLT-- -HEIMCSRCC DKKSCGNRNE
Xt COE1     ---------- ---------- ---------- ---------- ---------- ---------- ---------- ---------- ---------- ---------- ----------
Dr COE1     ALYDRQGQPV EIERTSFVGF VEKEK----A RIGEKTNNGI HYRLQLLYSN GIRTEQDFYV RLIDSMTKQA IIYEGQDKNP EMLTALFLSS SPSVSISRCC DKKSCGNRNE
COE2
Mm COE2     ALYDRQGQPV EIERTAFVDF VENDK----E QGNEKTNNGT HYKLQLLYSN GVRTEQDLYV RLIDSVTKQP IAYEGQNKNP EMCRVLLT-- -HEVMCSRCC EKKSCGNRNE
Hs COE2     ALYDRQGQPV EIERTAFVDF VENDK----E QGNEKTNNGT HYKLQLLYSN GVRTEQDLYV RLIDSVTKQP IAYEGQNKNP EMCRVLLT-- -HEVMCSRCC EKKSCGNRNE
Xt COE2     ALYDRQGQPI EIERTAFVDF VENEK----E LSTEKTNNGT HYKLQLLYSN GVRTEQDLYV RLIDSVTKQP IAYEGQNKNP EMCRVLLT-- -HEVMCSRCC EKKSCGNRNE
Dr COE2     ALYDRNGQPV EVERTSFVDF VEQDK----- -TGEKTNNGT HYKLQLLYSN GVRTEQDLYA RLIDSVTKQP ISYEGQNKNP EMCRVLLT-- -HEVMCSRCC EKKSCGNRNE
COE3
Mm COE3     ALYDRQGQPV EIERTAFVDF VEKEK----E PNNEKTNNGI HYKLQLLYSN GVRTEQDLYV RLIDSMTKQA IVYEGQDKNP EMCRVLLT-- -HEIMCSRCC DKKSCGNRNE
Hs COE3     ALYDRQGQPV EIERTAFVDF VEKEK----E PNNEKTNNGI HYKLQLLYSN GVRTEQDLYV RLIDSMTKQA IVYEGQDKNP EMCRVLLT-- -HEIMCSRCC DKKSCGNRNE
Xt COE3     ALYDRQGQPV EIERTAFVDF VEKDK----E PNSEKTNNGI HYKLQLLYSN GVRTEQDLYV RLIDSMTKQA IIYEGQDKNP EMCRVLLT-- -HEIMCSRCC DKKSCGNRNE
Dr COE3     ALYDRQGQPV EIERTAFVDF VEKEK----E PNSEKTNNGI HYKLQLLYSN GVRTEQDLYV RLIDSMTKQA IIYEGQDKNP EMCRVLLT-- -HEIMCSRCC DKKSCGNRNE
EBF4
Mm EBF4     AMYDRQGQPV EVERTAFIDF VEKDR----E PGTEKTNNGI HYRLRLVYNN GLRTEQDLYV RLIDSMSKQA IIYEGQDKNP EMCRVLLT-- -HEIMCSRCC DRKSCGNRNE
Hs EBF4     AMYDRQGQPV EVERTAFIDF VEKDR----E PGAEKTNNGI HYRLRLVYNN GLRTEQDLYV RLIDSMSKQA IIYEGQDKNP EMCRVLLT-- -HEIMCSRCC DRKSCGNRNE
Md EBF4     AMYDRQGQPV EIERTAFIDF VEKER----E PSGERTNNGI HYRLQLLYSN GIRTEQDLYV RLIDSMSKQA IIYEGQDKNP EMCRVLLT-- -HEIMCSRCC DKKSCGNRNE
Unassigned
DrCOE       ALYDSQGQSV EIEQTTFVDF VEKDK----E PVSEKTNNGI HYKLQLLYNN GVRTEQDLYI RLIDSVTKQA IVFEGQDKNP EMCRVLLT-- -HETMCSRCC DKKSCGNRNE
LAMPREYS
PmCOE-A     ALYDRQGQPV EIERTAFVDF VEKDR----E PNSEKTNNGI HYRLQLLYSN GVRTEQDLYV RLIDSMNKQX XXXXXXXXXX XXXXXXXX-- -XXXXXSRCC DKKNCGNRNE
NON-VERTEBRATES
Ci COE      ALYDRQGQPV EIERSAFVGF VENET----E IAGEKTNNGI QYRLQLLYHS GVRTEQDVFV RLIDSATKQS ITYEGQDKNP EMRRVLLT-- -HEIMCSRCC DKKSCGNRNE
Bf COE      ALYDRQGQPV EIERTAFIDF VEKDRISPQE TAGENTRNGI HYRLQLLYSN GVRTEQDLYV RLIDSMTKQA IVYEGQDKNP EMQRVLLT-- -HEIMCSRCC DKKSCGNRNE
Lg COE      ALYDRQGQPV EIERTAFIDF VEKDQ----E HESQKTNNGI HYRLQLLFAN GVRQEQEIYV RLIDSVSKQA IIYEGQDKNP EMCRVLLT-- -HEVMCSRCC DKKSCGNRNE
Cc COE      ALYDRQGQPV EVERTAFIDF VEKER----E PEG-KTNNGI HYRLQLLYAN GVRQEQDLYV RLIDSVTKQA IIYEGQDKNP EMCRCLLT-- -HEIMCSRCC DKKSCGNRNE
Dm COE      ALYDRAGQPI EIERTAFIGF IEKDS----E SDATKTNNGI QYRLQLLYAN GARQEQDIFV RLIDSVTKQA IIYEGQDKNP EMCRVLLT-- -HEVMCSRCC DKKSCGNRNE
Nv COE      AFFDHNGQAV EIERASFIDF IEHLP----E --DKNVRNGV LYRVLLLYSN GIRTEQDIFV RLIDAATKQI VPYEGQDKNP EMRRVLLT-- -HEVMCSRCC EKKSCGNKNE

                                    DBD
GNATHOSTOMES
COE1
Mm COE1     TPSDPVIIDR FFLKFFLKCN QNCLKNAGNP RDMRRFQVVV STTVNVDGHV LAVSDNMFVH NNSKHGRRAR RL-DPSEGT- PSYLEH---- ---------- ------ATPC
```

```
Hs COE1      TPSDPVIIDR FFLKFFLKCN QNCLKNAGNP RDMRRFQVVV STTVNVDGHV LAVSDNMFVH NNSKHGRRAR RL-DPSEGT- PSYLEH---- ---------- ------ATPC
Md COE1      TPSDPVIIDR FFLKFFLKCN QNCLKNAGNP RDMRRFQVVV STTVNVDGHV LAVSDNMFVH NNSKHGRRAR RL-DPSEGT- PSYLEH---- ---------- ------ATPC
Xt COE1      ---------- FFLKFFLKCN QNCLKNAGNP RDMRRFQVVV STTINVDGHV LAVSDNMFVH NNSKHGRRAR RL-DPSDGT- PSYLEHEA-- ---------- ------ATPC
Dr COE1      TPSDPVIIDR FFLKFFLKCN QNCLKNAGNP RDMRRFQVVV STTVSVDGHV LAVSDNMFVH NNSKHGRRAR RL-DPSEGT- PSYLEH---- ---------- ------ATPC
COE2
Mm COE2      TPSDPVIIDR FFLKFFLKCN QNCLKTAGNP RDMRRFQVVL STTVNVDGHV LAVSDNMFVH NNSKHGRRAR RL-DPSE--- ---------- ---------- ------ATPC
Hs COE2      TPSDPVIIDR FFLKFFLKCN QNCLKTAGNP RDMRRFQVVL STTVNVDGHV LAVSDNMFVH NNSKHGRRAR RL-DPSE--- ---------- ---------- ------ATPC
Xt COE2      TPSDPVIIDR FFLKFFLKCN QNCLKTAGNP RDMRRFQVVL STTVNVDGHV LAVSDNMFVH NNSKHGRRAR RL-DPSE--- ---------- ---------- ------ATPC
Dr COE2      TPSDPVIIDR FFLKFFLKCN QNCLKTAGNP RDMRRFQVVL STTVCVDGPV LAISDNMFVH NNSKHGRRSR RM-DPNETV- ENNMEY---- ---------- ------ATPC
COE3
Mm COE3      TPSDPVIIDR FFLKFFLKCN QNCLKNAGNP RDMRRFQVVV STTVNVDGHV LAVSDNMFVH NNSKHGRRAR RL-DPSE--- ---------- ---------- ------ATPC
Hs COE3      TPSDPVIIDR FFLKFFLKCN QNCLKNAGNP RDMRRFQVVV STTVNVDGHV LAVSDNMFVH NNSKHGRRAR RL-DPSEGTA PSYLEN---- ---------- ------ATPC
Xt COE3      TPSDPVIIDR FFLKFFLKCN QNCLKNAGNP RDMRRFQVVV STTVNVDGHV LAVSDNMFVH NNSKHGRRAR RL-DPSEGTA PSYLENV--- ---------- ------ATPC
Dr COE3      TPSDPVIIDR FFLKFFLKCN QNCLKNAGNP RDMRRFQVVV STTVNVDGHV LAVSDNMFVH NNSKHGRRAR RL-DPSEGTA TPYLENA--- ---------- ------ATPC
EBF4
Mm EBF4      TPSDPVIIDR FFLKFFLKCN QNCLKNAGNP RDMRRFQVVV STTVSVDGHV LAVSDNMFVH NNSKHGRRAR RL-DPSEA-- ---------- ---------- ------ATPC
Hs EBF4      TPSDPVIIDR FFLKFFLKCN QNCLKNAGNP RDMRRFQVVV STTVSVDGHV LAVSDNMFVH NNSKHGRRAR RL-DPSEA-- ---------- ---------- ------ATPC
Md EBF4      TPSDPVIIDR FFLKFFLKCN QNCLKNAGNP RDMRRFQVVV STTVSVDGHV LAVSDNMFVH NNSKHGRRAR RL-DPSEA-- ---------- ---------- ------ATPC
Unassigned
DrCOE        TPSDPVIIDR FFLKFFLKCN QNCLKNAGNP RDTRRFQVLV STTASVKAHI LAISDNIFVH NNSKHGRRAR RF-ELIEA-- ---------- ---------- ------EIPC
LAMPREYS
PmCOE-A      TPSDPVIIDR FFLKFFLKCN QNCLKNAGNP RDMRRFQVVV STTVHVDGHV LAVSDNMFVH NNSKHGRRAR RI-DPSE--- ---------- ---------- ------ATPC
NON-VERTEBRATES
Ci COE       TPSDPVVIDR YFLKFFLKCN QNCLKNAGNP RDMRRFQVVV STTVHVDGHV LAVSDNMFVH NNSKHGRRAR RV-DPSE--- ---------- ---------- ------ASPT
Bf COE       TPSDPVIIDR FFLKAFLKCN QNCLKNAGNP RDMRRFQVVI ATTVNVDGHV LAVSDNMFVH NNSKHGRRAR RL-DPSEDGE MSFCGDSPNI NRGQK----- ------ATPC
Lg COE       TPSDPVIIDR FFLKFFMKCN QNCLKNAGNP RDMRRFQVAV STSVAIDGPL LTISDNMFVH NNSKHGRRAR RL-DPTEGGD LRFSHLRPKL SPFAA----- ------TTPC
Cc COE       TPSDPVIIDR FFLKFFMKCN QNCLKNAGNP RDMRRFQVSI GTSSSVEGPL LAVSDNMFVH NNSKHGRRAR RL-DPGDAGF LCA------- ---------- ------ATPT
Dm COE       TPSDPVIIDR FFLKFFLKCN QNCLKNAGNP RDMRRFQVVI STQVAVDGPL LAISDNMFVH NNSKHGRRAK RL-DTTEGTG NTSLSISGHP LAPDSTYDGL YPPLPVATPC
Nv COE       TPSDPVVVDR YCLKFFMKCN QNCLKNAGNP KDMRRFQVHV STSVDPMYGM IACSDNMFVH NNSKHGRRTR SRADGNEQDT SA-------- ---------- ------DDPC
```

```
                                                 IPT                                               H1
GNATHOSTOMES
COE1
Mm COE1      IKAISPSEGW TTGGATVIII GDNFFDGLQV IFGTMLVWSE LITPHAIRVQ TPPRHIPGVV EVTLSYKSKQ FCKGTPGRFI YT-ALNEPTI DYGFQRLQKV IPRHPGDPER
Hs COE1      IKAISPSEGW TTGGATVIII GDNFFDGLQV IFGTMLVWSE LITPHAIRVQ TPPRHIPGVV EVTLSYKSKQ FCKGTPGRFI YT-ALNEPTI DYGFQRLQKV IPRHPGDPER
Md COE1      IKAISPSEGW TTGGATVIII GDNFFDGLQV IFGTMLVWSE LITPHAIRVQ TPPRHIPGVV EVTLSYKSKQ FCKGTPGRFI YT-ALNEPTI DYGFQRLQKV IPRHPGDPER
Xt COE1      IKAISPSEGW TTGGATVIVI GDNFFDGLQV IFGTMLVWSE LITPHAIRVQ TPPRHIPGVV EVTLSYKSKQ FCKGTPGRFI YT-ALNEPTI DYGFQRLQKV IPRHPGDPER
Dr COE1      IKAISPSEGW TTGGATVIII GDNFFDGLQV IFGTMLVWSE LITPHAIRVQ TPPRHIPGVV EVTLSYKSKQ FCKGTPGRFI YT-ALNEPTI DYGFQRLQKV IPRHPGDPER
COE2
Mm COE2      IKAISPSEGW TTGGAMVIII GDNFFDGLQV VFGTMLVWSE LITPHAIRVQ TPPRHIPGVV EVTLSYKSKQ FCKGAPGRFI YT-ALNEPTI DYGFQRLQKV IPRHPGDPER
Hs COE2      IKAISPSEGW TTGGAMVIII GDNFFDGLQV VFGTMLVWSE LITPHAIRVQ TPPRHIPGVV EVTLSYKSKQ FCKGAPGRFI YT-ALNEPTI DYGFQRLQKV IPRHPGDPER
Xt COE2      IKAISPSEGW TTGGAMVIII GDNFFDGLQV VFGTMLVWSE LITPHAIRVQ TPPRHIPGVV EVTLSYKSKQ FCKGAPGRFI YT-ALNEPTI DYGFQRLQKV IPRHPGDPER
Dr COE2      IKAISPSEGW TTGGAMVIVI GENFFDGLQV VFGSMLVWSE LITPHAIRVQ TPPRHIPGVV EVTLSYKSKQ FCKGAPGRFI YT-ALNEPTI DYGFQRLQKL IPRHPGDPDK
COE3
Mm COE3      IKAISPSEGW TTGGATVIII GDNFFDGLQV VFGTMLVWSE LITPHAIRVQ TPPRHIPGVV EVTLSYKSKQ FCKGAPGRFV YT-ALNEPTI DYGFQRLQKV IPRHPGDPER
Hs COE3      IKAISPSEGW TTGGATVIII GDNFFDGLQV VFGTMLVWSE LITPHAIRVQ TPPRHIPGVV EVTLSYKSKQ FCKGAPGRFV YT-ALNEPTI DYGFQRLQKV IPRHPGDPER
Xt COE3      IKAISPSEGW TTGGATVIII GDNFFDGLQV VFGTMLVWSE LITPHAIRVQ TPPRHIPGVV EVTLSYKSKQ FCKGAPGRFV YT-ALNEPTI DYGFQRLQKV IPRHPGDPER
Dr COE3      IKAISPSEGW TTGGATVIII GDNFFDGLQV VFGTMLVWSE LITPHAIRVQ TPPRHIPGVV EVTLSYKSKQ FCKGAPGRFV YT-ALNEPTI DYGFQRLQKV IPRHPGDPER
EBF4
Mm EBF4      IKAISPGEGW TTGGATVIII GDNFFDGLQV VFGNVLLWSE LITPHAIRVQ TPPRHIPGVV EVTLSYKSKQ FCKGAPGRFV YT-ALNEPTI DYGFQRLQKV IPRHPGDPER
Hs EBF4      IKAISPGEGW TTGGATVIVI GDNFFDGLQV VFGNVLVWSE LITPHAIRVQ TPPRHIPGVV EVTLSYKSKQ FCKGCPGRFV YT-ALNEPTI DYGFQRLQKV IPRHPGDPER
Md EBF4      IKAISPSEGW TTGGATVIVI GDNFFDGLQV VFGTMLVWSE LITPHAIRVQ TPPRHIPGVV EVTLSYKSKQ FCKGAPGRFV YT-ALNEPTI DYGFQRLQKV IPRHPGDPER
Unassigned
Dr COE       IKAISPSEGW TSGGASVIII GDQFFSGLQV VFGTMLVWSE VITPHAIRVQ TPPRHIPGVV EVTLSYKSKQ FCRGAPGRFV YT-ALNEPTI DYGFQRLQKV IPHHPGDVER
LAMPREYS
PmCOE-A      IKAISPSEGW TTGGATVIII GDNFFDGLQV VFGTMLVWSE LITPHAIRVQ TPPRHIPGVV EVTLSYKSKQ FCKGAPGRFV YT-ALNEPTI DYGFQRLQKL IPRHPGDPER
NON-VERTEBRATES
Ci COE       IKAINPAEGW TTGGATVVIV GENFFDGLQV VFGSMVVWSE LITQHAIRVQ TPPRHLPGVV EVTLSYKNKQ FCSGAPGRFV YT-ALNEPTL DYGFQRLLKT VPRHPGDPER
Bf COE       IKAISPSEGW TTGGATVIII GDNFFDGLQV VFGTMLVWSE LITPHAIRVQ TPPRHIPGVV EVTLSYKSKQ FCKGAPGRFV YT-SLNEPTI DYGFQRLAKL VPRHPGDPER
Lg COE       IKAICPSEGW TSGGTTVIII GDNFFDGLQL VFGTMLVWSE LLTSHAIRVQ TPPRHIPGVV EVTLSYKSKQ FCKGAPGRFV YT-ALTEPTI DYGFQRLMKL VPRHPGDPEK
Cc COE       IKAICPSEGW TTGGTTVIII GDNFFDGLQV VFGTMLVWSE LITSHAIRVQ TPPRHIPGVV EVTLSYKSKQ FCKGAPGRFV YT-ALTEPTI DYGFQRLMKL VPRHPGDPER
Dm COE       IKAISPSEGW TTGGATVIIV GDNFFDGLQV VFGTMLVWSE LITSHAIRVQ TPPRHIPGVV EVTLSYKSKQ FCKGSPGRFV YVSALNEPTI DYGFQRLQKL IPRHPGDPEK
Nv COE       IKAICPNEGW TIGGSNVILI GDNFFDGLQV VFGSFIVWSE FITPHALRVQ APPSPMPGVV KVYLMHKEKQ YCKYAPAKFG YT-ALVEPTI DYGFQRLSKL IPRHPGDPER

                       H2d                H2a
GNATHOSTOMES
COE1
Mm COE1      LPKEVILKRA ADLVEALYGM PHNNQ--EII LKRAADIAEA LYSVPRNHNQ LPALA-NTSV HA-------- ---------- ------GMMG VNS------F SGQLAVNVSE
Hs COE1      LPKEVILKRA ADLVEALYGM PHNNQ--EII LKRAADIAEA LYSVPRNHNQ LPALA-NTSV HA-------- ---------- ------GMMG VNS------F SGQLAVNVSE
Md COE1      LPKEVILKRA ADLVEALYGM PHNNQ--EII LKRAADIAEA LYSVPRNHNQ LPALA-NTSV HA-------- ---------- ------GMMG VNS------F SGQLAVNVSE
Xt COE1      LPKEVILKRA ADLVEALYGM PHNNQ--EII LKRAADIAEA LYSVPRNHSQ LPALT-NSSV HP-------- ---------- ------GMMG VNS------F SGQLAVNVSE
Dr COE1      LPKEVILKRA ADLVEALYGM PHNNQ--EII LKRAADIAEA LYNVPRGHNQ LPGLT-NSSV HS-------- ---------- ------GMMG VNS------F HSQLAVNVSD
COE2
Mm COE2      LAKEMLLKRA ADLVEALYGT PHNNQ--DII LKRAADIAEA LYSVPRNPSQ IPALS-SSPA HS-------- ---------- ------GMMG INS------Y GSQLGVSISE
Hs COE2      LAKEMLLKRA ADLVEALYGT PHNNQ--DII LKRAADIAEA LYSVPRNPSQ LPALS-SSPA HS-------- ---------- ------GMMG INS------Y GSQLGVSISE
Xt COE2      LAKEMLLKRA ADLVEALYGT PHNNQ--DII LKRAADIAEA LYSVPRNHNQ IPALS-SSPV HS-------- ---------- ------GMMG INS------Y GGQLGVSISE
Dr COE2      LAKEMLLKRA ADVVESLYGN TTSNQ--DML LKRAADIAEA LYSVPRPHSQ LQAMP-SSPV HG-------- ---------- ------SVMG LSS------Y PTQLGVSIGE
COE3
Mm COE3      LPKEVLLKRA ADLVEALYGM PHNNQ--EII LKRAADIAEA LYSVPRNHNQ IPTLG-NTPA HT-------- ---------- ------GMMG VNS------F SSQLAVNVSE
Hs COE3      LPKEVLLKRA ADLVEALYGM PHNNQ--EII LKRAADIAEA LYSVPRNHNQ IPTLG-NNPA HT-------- ---------- ------GMMG VNS------F SSQLAVNVSE
Xt COE3      LPKEVLLKRA ADLVEALYGM PHNNQAKEII LKRAADIAEA LYSVPRNHNQ IPSLA-NTPS HS-------- ---------- ------GMMG VNS------F SSQLAVNVSE
Dr COE3      LPKEVLLKRA ADLVEALYGM PHNNQ--EII LKRAADIAEA LYSVPRNHNQ IPSLA-NTAS HG-------- ---------- ------GMMG VNS------F SSQLAVNVSE
EBF4
Mm EBF4      LPKEVLLKRA ADLAEALYGV PSSNQ--ELL LKRAADVAEA LYSAPRAPAP LGPLA-PSHP HP-------- ---------- ------AVVG INA------F SSPLAIAVGD
Hs EBF4      LPKEVLLKRA ADLAEALYGV PGSNQ--ELL LKRAADVAEA LYSTPRAPGP LAPLA-PSHP HP-------- ---------- ------AVVG INA------F SSPLAIAVGD
Md EBF4      LPKEVLLKRA ADLVEALYGM PHSNQ--DLI IKRAADIAEA LYSVPRTPSQ LGSLA-PGHP HT-------- ---------- ------AMMG INS------F GSQLAVNIGD
Unassigned
DrCOE        LPKEVLLKRA ADLIETFYGA PQNNQ--EII LKRASDIAEA LNSIPRNPS- ------HTNP H--------- ---------- ------GMMA VNS------Y DGQLSLNTEV
LAMPREYS
PmCOE-A      LPK------- ---------- ----Q--EII LKRAADIAEA LYSVPRNHNQ LPAIN-NSPP GLP------- ---------- ------GMMG MNS------F GGQLAISVSE
NON-VERTEBRATES
Ci COE       LPK------- ---------- -------EII LKRAADVMEA VISRQYAPPS QMPPSAGITP PAPHLAAAPC APPGSFVPQS ASAA--MAVA MNGYAAAA-V SSQFGGTPDR
Bf COE       LPK------- ---------- -------EII LKRAADLAEA IYSMPRNPNL PALTGPRSPA VNNSVGAG-- ---------- ------GMMG MNS------F GNQLAVSVPD
Lg COE       LPK------- ---------- -------EII LKRAADLAEA LYSMPRNHNQ LSLPAPRSPA MNNTS----- ---------- ------PMSG FNA------Y PSQLAVSVGS
Cc COE       LPKGICEGHV GPLSSQIFLS CPQ----EII LKRAADLAEA LYSMPRNPNQ LSLPAPRSPA MN-------- ---------- ------NTTG FNS------Y TGQLAVSVQD
Dm COE       LQK------- ---------- -------EII LKRAADLVEA LYSMPRSPG- ---------- ---------- ---------- ------GSTG FNS------Y AGQLAVSVQD
Nv COE       IPK------- ---------- -------EIV LKRAADLAET LYQMPRTPTQ VHPSQYASTG LATPKSPALV GGQHYVTLLP DSMTNVFTQS ITQDNQPIAY SSHNDNASDQ

GNATHOSTOMES
COE1
Mm COE1      ASQATNQ--- ---------- ---------- --GFTR-NSS SV-------- SPHG-YV-P- STTPQQTN-- ---------- YNS-VTTSMN GYGSAA-MSN LG--GSPTFL
```

```
Hs COE1      ASQATNQ--- ---------- ---------- --GFTR-NSS SV-------- SPHG-YV-P- STTPQQTN-- ---------- YNS-VTTSMN GYGSAA-MSN LG--GSPTFL
Md COE1      ASQATNQ--- ---------- ---------- --GFTR-NSS SV-------- SPHG-YV-P- STTPQQTN-- ---------- YNS-VTTSMN GYGNAG-MSN LG--GSPTFL
Xt COE1      PSQVTNQ--- ---------- ---------- --GFSR-NTS SV-------- SPHS-YA-P- STTPQQTN-- ---------- YSS-VTTSMN GYGNTA-MSN LA--GSPSFL
Dr COE1      STQAANQ--- ---------- ---------- --GFSR-NTS SV-------- SPHG-YV-P- STTPQQSS-- ---------- YST-VSTSMN GYGNAG-MTT LG--GSPNFL
COE2
Mm COE2      STQGNNQ--- ---------- ---------- --GYIR-NTS SI-------- SPRG-YS-S- SSTPQQSN-- ---------- YST-SSNSMN GYSNVP-MAN LGVPGSPGFL
Hs COE2      STQGNNQ--- ---------- ---------- --GYIR-NTS SI-------- SPRG-YS-S- SSTPQQSN-- ---------- YST-SSNSMN GYSNVP-MAN LGVPGSPGFL
Xt COE2      SQAN-NQ--- ---------- ---------- --GYIR-NTS SI-------- SPRG-YS-S- SSTPQQSN-- ---------- YST-PSNSMN GYSNVP-MSN LGVPGSPGFI
Dr COE2      PGQTSGQ--- ---------- ---------- --GYTR-NSS SL-------- SPRG-YP-S- SSTPQQSA-- ---------- YGS-NGGM-- SYGAVP-MSS LGVSGSPGFN
COE3
Mm COE3      TSQANDQV-- ---------- ---------- --GYSR-NTS SV-------- SPRG-YV-P- SSTPQQSN-- ---------- YNT-VSTSMN GYGSGA-MAN LGVPGSPGFL
Hs COE3      TSQANDQV-- ---------- ---------- --GYSR-NTS SV-------- SPRG-YV-P- SSTPQQSN-- ---------- YNT-VSTSMN GYGSGA-MAS LGVPGSPGFL
Xt COE3      TSQANDQV-- ---------- ---------- --GYSR-NTS SV-------- SPRG-YV-P- SSTPQQSN-- ---------- YNT-VSNSMN GYGNAG-MPN LGVPGSPGFL
Dr COE3      TSQVFLSTV- ---------- ---------- --GYSR-NTS SV-------- SPRG-YV-P- SSTPQQSN-- ---------- YNT-VSNSMN GYGNTG-MPN LGVPSSPGFL
EBF4
Mm EBF4      TTP--EP--- ---------- ---------- --GYAR-SCG SA-------- SPR--FA-P- SPGSQQSS-- ---------- YGSGLGAGLG SYGAPG-VTG LGVPGSPSFL
Hs EBF4      ATPGPEP--- ---------- ---------- --GYAR-SCS SA-------- SPRG-FA-P- SPGSQQSG-- ---------- YGGGLGAGLG GYGAPG-VAG LGVPGSPSFL
Md EBF4      STQGL----- ---------- ---------- --GYSR-NTS SV-------- SPRG-YV-P- SSTPQQSS-- ---------- YSS-ITSSIN GYGATG-MAG LGVPSSPSFL
Unassigned
DrCOE        SQQDAERA-- ---------- ---------- --SFNR-S-- ---------- SNTG-FI-E- GSTSQQSE-- ---------- FNN-TNMCMN G......... ..........
LAMPREYS
PmCOE-A      ASQAADQ--- ---------- ---------- --GYTR-NSS SV-------- SPHG-YV-P- SSTPQQSH-- ---------- YSS-VSSSMN GYGVSGGMGG LG........
NON-VERTEBRATES
Ci COE        FD-TGSDS-- ---------- ---------- --GYSR--GN SV-------- SPRNGYS-P- QTTPHSLNSG SIGSMVGLTT VGA-VPAPAP YHCAPS-FNS YSSASVLKYN
Bf COE        NSSTNGNGQV QGFVPVFTYS GLKEDCAEQT NHGYSR-QSN SV-------- SPRG-YG-A- -STPHSTN-G SISSYST--- ----SASGLN GYGSSG-NLS NMPVPSSPGF
Lg COE        VA-DTASGQW -EEGWCLKRT SR-------- --SYNRSQSS SV-------- SPRG-YG-SN GSTPHSNG-S YT-------- ----STPAMN GYHHGG-GAL GNMVNAPHSP
Cc COE        TA-N---GQW -EE------- ---------- --GWSH-LTS SLLGYSSRMT PPHG-YP-M- CSSGISTS-C SAYSGSS--- ----STYSRS GYIDGC-TSS IPIPGTPHSV
Dm COE        GS-----GQW TED------- ---------- --DYQRAQSS SV-------- SPRGGYCSS- ASTPHSSG-G SY-------- ---------- --GATA-ASA AVAATANGYA
Nv COE        TD-MNNNVVQ SS-------- ---------- --NSSR-LTS NA-------- TLSG-VT-N- MSNHDSHHNG VMASSDEGLS AIATVSNSCY GNGSPA-LSC MAVPASPGYF


GNATHOSTOMES
COE1
Mm COE1      NGSAANSPYA IVPSSPTMA- -SSTSLPSNC SSSSGIFSFS PANMVSAVKQ KSA--FAPVV RPQTS-PPPT CTST-NGNSL QAISGMIVPP M
Hs COE1      NGSAANSPYA IVPSSPTMA- -SSTSLPSNC SSSSGIFSFS PANMVSAVKQ KSA--FAPVV RPQTS-PPPT CTST-NGNSL QAISGMIVPP M
Md COE1      NGSAANSPYA IVPSSPTMA- -SSTSLPSNC SSSSGIFSFS PANMVSAVKQ KSA--FAPVV RPQTS-PPPT CTST-NGNSL QAISGMIVPP M
Xt COE1      NGSAANSPYA IVPSSPTMA- -SSTSLPSNC SSSSGIFSFS PANMVSAVKQ KSA--FAPVV RPQAS-PPPT CSSS-SATSL QAISGMIAPP M
Dr COE1      NGSAANSPYA IVPSSPTMA- -SSTSLPSNC SSSSGIFSFS PANMVSAVKQ KSA--FAPVV RPQAS-PPPT CTSA-NGNGL QGESG----- -
COE2
Mm COE2      NGSPTGSPYG IMSSSPTV-- --------GS SSTSSILPFS SSVFP-AVKQ KSA--FAPVI RPQGS-PSPA CSSG-NGNGF RAMTGLVVPP M
Hs COE2      NGSPTGSPYG IMSSSPTV-- --------GS SSTSSILPFS SSVFP-AVKQ KSA--FAPVI RPQGS-PSPA CSSG-NGNGF RAMTGLVVPP M
Xt COE2      NGSPTTSPYG IMPSSPPV-- --------GS SGSSSILPFS SSVFP-SIKQ KSA--FAPVI RPQGS-PSPA CSSS-NSNGF RAMTGLVVPP M
Dr COE2      SASPNSSPYA IMPSSPP--- --------GS SSSSSLLPFS S--FPSSTKQ KSA--FAPVL RPQGF-PHHP SAKTSGGTSF RAMTGLVVPP M
COE3
Mm COE3      NGSSANSPYG ---------- ---------- ---------- -------MKQ KSA--FAPVV RPQAS-PPPS CTSA-NGNGL QAMSGLVVPP M
Hs COE3      NGSSANSPYG IVPSSPTMAA -SSVTLPSNC SSTHGIFSFS PANVISAVKQ KSA--FAPVV RPQAS-PPPS CTSA-NGNGL QAMSGLVVPP M
Xt COE3      NGSSANSPYG IVPSSPTMAA -SSVTLPSNC SSTHGIFSFS PANVISAVKQ KSA--FAPVV RPQAS-PPPS CTSA-NGNGL QDMYFNPTFP KS
Dr COE3      NGSSANSPYG IVPSSPTMAA -SSVSLSSNC SSTHGIFSFS PANVISAVKQ KSA--FAPVV RPQAS-PPPS CTSA-NGNGL QAMSGLVVPP M
EBF4
Mm EBF4      NGSTATSPFA IMPSSPPLAA ASSMSLPAAA PTTS-VFSFS PVNMICAVKQ RSA--FAPVL RPPSS-PSQA CPRA-HREGL PAQRTGR
Hs EBF4      NGSTATSPFA IMPSSPPLAA ASSMSLPAAA PTTS-VFSFS PVNMISAVKQ RSA--FAPVL RPPSS-PPQA CPRA-HGEGL PDQSFEDSDK FHSPARGLQG LAYS
Md EBF4      NGSTANSPYA IMPSSPPL-A ASSISLPAAA PTTS-VFSFS PVNMISAVKQ KSA--FAPPA SLLCP.PLVP SVPL.LLDTD PA..FEDSDK FHSPPGALQG LAYS
Unassigned
DrCOE        .......... .......... .......... .......... .......... .......... .......... .......... ..........
LAMPREYS
PmCOE.A      .......... .......... .......... .......... .......... .......... .......... .......... ..........
NON-VERTEBRATES
Ci COE        LFTVIPPSPH NGMNPLPSSG TTP------- ----GIFSFS PANMISAAKQ KSA--FAPVH RPHNS-PSPL APSNGNIGKI SCFVLSSI
Bf COE        LNGSTIPSSP TMPPTPSSLA TNASTP---- ----GIFFFS PANMISAVKQ KSA--FAPVV RPQ---SSPS PAGASSNGTG LQGGWTPTIP SAVC
Lg COE        PMHNIGSQGL GSCTP----- ---------- ----GIFSFS PANMITAVKQ RSA--FAPVL RGGNS-PPPT FPGIQTSGWA HPLIHVSIIS TSLPSNALKV SY
Cc COE        PSQSPSLPPT ASCSVAP--- ---------- ----GLHSFS HANVLSAVKQ RSA--FAPVL RPGSSSPVPG MTSIAAAGWA HTLPTSGMTD CHSIHA
Dm COE        PAPNM----- ---------- ---------- ----GTLSSS PGSVFNSTSR VSSLSFNPFA LP-------- --TCNTQGYS TQLVTSTK
Nv COE        TGAFIPQSPS LPPTPNSVPP SSNN------ ----SIFSFP P-NMIQAVKQ KSA--FNAVS RGPESTAHPR SPAVVQGVMT STYNGPFAVP VSSVNAYIKS IQPQVKP
```

Figure S1 : Alignment used in the phylogenetic analysis of metazoan COE proteins

Dashes indicate missing residues; dots at the C-terminal end of DrCOE and PmCOE-A stand for undetermined residues. Only the positions shaded in yellow were used in the phylogenetic analysis. Highly truncated (XtCOE1, PmCOE-B) or divergent (Ce COE) predicted sequences were excluded from the analysis. The 8 to 10 residues inclusion between the DBD and IPT domains in vertebrate COE proteins, due to the existence of an alternative splice donor site is underlined in MmCOE1. Same abbreviations as in Figure 1. DBD, DNA Binding Domain ; IPT, Ig-like/Plexin/transcription Factor Domain ; H1/2d/2a, Helix H1/2d/2a.
