# Supplementary material for: The metazoan history of the COE transcription factors. Selection of a variant HLH motif by mandatory inclusion of a duplicated exon in vertebrates
Source: BMC Evol Biol. 2008 May 2;8:131. doi: 10.1186/1471-2148-8-131 (PMC2394523; doi:10.1186/1471-2148-8-131)
Supplement: Additional file 2 — Sequence alignments used in the phylogenetic analysis of metazoan COE proteins. [file 1471-2148-8-131-S2.pdf]

DNA BINDING DOMAIN

**GNATHOSTOMES**											
COE1											
M. musculus	COE1	RAHFEKQPPS	NLRKSNFFHF	VLALYDRQGQ	PVEIERTAFV	GFVEKEK---	-EANSEKTNN	GIHYRLQLLY	SNGIRTEQDF	YVRLIDSMTK	QAIVYEGQDK
H. sapiens	COE1	RAHFEKQPPS	NLRKSNFFHF	VLALYDRQGQ	PVEIERTAFV	GFVEKEK---	-EANSEKTNN	GIHYRLQLLY	SNGIRTEQDF	YVRLIDSMTK	QAIVYEGQDK
M. domestica	COE1	RAHFEKQPPS	NLRKSNFFHF	VLALYDRQGQ	PVEIERTAFV	GFVEKEK---	-EANSEKTNN	GIHYRLQLLY	SNGIRTEQDF	YVRLIDSMTK	QAIVYEGQDK
O. anatinus	COE1	RAHFEKQPPS	NLRKSNFFHF	VLALYDRQGQ	PVEIERTAFV	GFVEKEK---	-EANSEKTNN	GIHYRLQLLY	SNGXXXXXXX	XXXXXXXXXX	XXXXXXXXXX
G. gallus	COE1	RAHFEKQPPS	NLRKSNFFHF	VLALYDRQGQ	PVEIERTAFV	GFVEKEK---	-EANSEKTNN	GIHYRLQLLY	SNGIRTEQDF	YVRLIDSMTK	QAIVYEGQDK
X. tropicalis	COE1	XXXXXXXXXX	XXXXXXXXXX	XXXXXXXXXX	XXXXXXXXXX	XXXXXXXXXX	XXXXXXXXXX	XXXXXXXXXX	XXXXXXXXXX	XXXXXXXXXX	XXXXXXXXXX
D. rerio	COE1	RAHFEKQPPS	NLRKSNFFHF	VLALYDRQGQ	PVEIERTSFV	GFVEKEK---	-ARIGEKTNN	GIHYRLQLLY	SNGIRTEQDF	YVRLIDSMTK	QAIIYEGQDK
COE2											
M. musculus	COE2	RAHFEKQPPS	NLRKSNFFHF	VLALYDRQGQ	PVEIERTAFV	DFVENDK---	-EQGNEKTNN	GTHYKLQLLY	SNGVRTEQDL	YVRLIDSVTK	QPIAYEGQNK
H. sapiens	COE2	RAHFEKQPPS	NLRKSNFFHF	VLALYDRQGQ	PVEIERTAFV	DFVENDK---	-EQGNEKTNN	GTHYKLQLLY	SNGVRTEQDL	YVRLIDSVTK	QPIAYEGQNK
O. anatinus	COE2	XXXXXXXXXX	XXXXXXXXXX	XXXXXXXXXX	XXXXXXXXXX	XXXXXXXXXX	XXXXXXXXXX	XXXXXXXXXX	XXXXXXXXXX	XXXXXXXXXX	XXXXXXXXXX
G. gallus	COE2	RAHFEKQPPS	NLRKSNFFHF	VLALYDRQGQ	PVEIERTAFV	DFVENDK---	-EQGNEKTNN	GTHYKLQLLY	SNGVRTEQDL	YVRLIDSVTK	QPITYEGQNK
X. tropicalis	COE2	RAHFEKQPPS	NLRKSNFFHF	VLALYDRQGQ	PIEIERTAFV	DFVENEK---	-ELSTEKTNN	GTHYKLQLLY	SNGVRTEQDL	YVRLIDSVTK	QPIAYEGQNK
D. rerio	COE2	RAHFEKQPPS	NLRKSNFFHF	VLALYDRNGQ	PVEVERTSFV	DFVEQDK---	---TGEKTNN	GTHYKLQLLY	SNGVRTEQDL	YARLIDSVTK	QPISYEGQNK
COE3											
M. musculus	COE3	RAHFEKQPPS	NLRKSNFFHF	VLALYDRQGQ	PVEIERTAFV	DFVEKEK---	-EPNNEKTNN	GIHYKLQLLY	SNGVRTEQDL	YVRLIDSMTK	QAIVYEGQDK
H. sapiens	COE3	RAHFEKQPPS	NLRKSNFFHF	VLALYDRQGQ	PVEIERTAFV	DFVEKEK---	-EPNNEKTNN	GIHYKLQLLY	SNGVRTEQDL	YVRLIDSMTK	QAIVYEGQDK
O. anatinus	COE3	RAHFEKQPPS	NLRKSNFFHF	VLALYDRQGQ	PVEIERTAFV	DFVEKEK---	-EPNNEKTNN	GIHYKLQLLY	SNGVRTEQDL	YVRLIDSMTK	QAIVYEGQDK
G. gallus	COE3	XXXXXXXXXX	XXXXXXXXXX	XXXXXXXXXX	XXXXXXXXXX	XXXXXXXXXX	XXXXXXXXXX	XXXXXXXXXX	XXXXXXXXXX	XXXXXXXXXX	XXXXXXXXXX
X. tropicalis	COE3	RAHFEKQPPS	NLRKSNFFHF	VLALYDRQGQ	PVEIERTAFV	DFVEKDK---	-EPNSEKTNN	GIHYKLQLLY	SNGVRTEQDL	YVRLIDSMTK	QAIIYEGQDK
D. rerio	COE3	RAHYEKQPPS	NLRKSNFFHF	VLALYDRQGQ	PVEIERTAFV	DFVEKEK---	-EPNSEKTNN	GIHYKLQLLY	SNGVRTEQDL	YVRLIDSMTK	QAIIYEGQDK
EBF4											
M. musculus	EBF4	RAHFEKQPPS	NLRKSNFFHF	VLAMYDRQGQ	PVEVERTAFI	DFVEKDR---	-EPGTEKTNN	GIHYRLRLVY	NNGLRTEQDL	YVRLIDSMSK	QAIIYEGQDK
H. sapiens	EBF4	RAHFEKQPPS	NLRKSNFFHF	VLAMYDRQGQ	PVEVERTAFI	DFVEKDR---	-EPGAEKTNN	GIHYRLRLVY	NNGLRTEQDL	YVRLIDSMSK	QAIIYEGQDK
M. domestica	EBF4	RAHFEKQPPS	NLRKSNFFHF	VLAMYDRQGQ	PVEIERTAFI	DFVEKER---	-EPSGERTNN	GIHYRLQLLY	SNGIRTEQDL	YVRLIDSMSK	QAIIYEGQDK
O. anatinus	EBF4	RAHFEKQPPA	NLRKSNFFHF	VLAMYDRHGQ	PVEVERSAFV	GFVEKGR---	-EHGGEKTNN	GIHYRLQLLY	SNGIRTEQDL	YVRLIDSVSK	QAIIYEGQDK
UNASSIGNED											
D. rerio	COE	RAHFEKQPPA	NLRKSNFFHF	VLALYDSQGQ	SVEIEQTTFV	DFVEKDK---	-EPVSEKTNN	GIHYKLQLLY	NNGVRTEQDL	YIRLIDSVTK	QAIVFEGQDK
**LAMPREYS**											
P. marinus	COE-A	RAHFEKQPPS	NLRKSNFFHF	VLALYDRQGQ	PVEIERTAFV	DFVEKDR---	-EPNSEKTNN	GIHYRLQLLY	SNGVRTEQDL	YVRLIDSMNK	QXXXXXXXXX
P. marinus	COE-B	XXXXXXXXXX	XXXXXXXXXX	XXXXXXXXXX	XXXXXXXXXX	XXXXXXXXXX	XXXXXXXXXX	XXXXXXXXXX	XXXXXXXXXX	XXXXXXXXXX	XXXXXXXXXX
**NON-VERTEBRATES**											
C. intestinalis	COE	RAHFEKQPPN	NLRKSNFFHF	VLALYDRQGQ	PVEIERSAFV	GFVENET---	-EIAGEKTNN	GIQYRLQLLY	HSGVRTEQDV	FVRLIDSATK	QSITYEGQDK
B. floridae	COE	RAHFEKQPPS	NLRKSNFFHF	VIALYDRQGQ	PVEIERTAFI	DFVEKDRISP	QETAGENTRN	GIHYRLQLLY	SNGVRTEQDL	YVRLIDSMTK	QAIVYEGQDK
S. purpuratus	COE	RAHFEKQPPS	NLRKSNFFHF	VIALYDRAGQ	PIEVERTSFV	DFIEKER---	-EPDATKTNN	GIHYRIQMLF	HNGVRTEQDL	YVRLIDSMTK	QAIIYEGQDK
L. gigantae	COE	RAHFEKQPPS	NLRKSNFFHF	VLALYDRQGQ	PVEIERTAFI	DFVEKDQ---	-EHESQKTNN	GIHYRLQLLF	ANGVRQEQEI	YVRLIDSVSK	QAIIYEGQDK
C. capitata	COE	RAHYEKQPPS	NLRKSNFFHF	VLALYDRQGQ	PVEVERTAFI	DFVEKER---	-EPEG-KTNN	GIHYRLQLLY	ANGVRQEQDL	YVRLIDSVTK	QAIIYEGQDK
D. melanogaster	COE	RAHFEKQPPS	NLRKSNFFHF	VIALYDRAGQ	PIEIERTAFI	GFIEKDS---	-ESDATKTNN	GIQYRLQLLY	ANGARQEQDI	FVRLIDSVTK	QAIIYEGQDK
C. elegans	COE	RAHFEKHPPN	NLRKSNFFHF	VIALYDRNSQ	PIEVERTQFA	GFVEKEK---	-EVDGQDTRN	GIHYRLSLMF	QNGIRSEHDL	FVRLIDSSTK	QAITYEGQDK
N. vectensis	COE	RCHFEKQPPA	NLRKSNFFHF	VLAFFDHNGQ	AVEIERASFI	DFIEH-----	-LPEDKNVRN	GVLYRVLLLY	SNGIRTEQDI	FVRLIDAATK	QIVPYEGQDK
A. queenslandica	COE	AAQFEKQPPD	NMRKSNFFHF	IISLFDGQQH	RIQVQKAVFK	DFYDTV----	-GTDEQEYRN	GLIYKLLVVY	SDGTQKEEEL	CVRLIDSNTK	QLVPYEGTNK

DNA BINDING DOMAIN

```
GNATHOSTOMES
COE1
MmCOE1       NPEMCRVLLT ---HEIMCSR CCDKKSCGNR NETPSDPVII D--RFFLKFF LKCNQNCLKN AGNPRDMRR- FQVVVSTTVN VDGHVLAVSD NMFVHNNSKH GRRARRL-DP
HsCOE1       NPEMCRVLLT ---HEIMCSR CCDKKSCGNR NETPSDPVII D--RFFLKFF LKCNQNCLKN AGNPRDMRR- FQVVVSTTVN VDGHVLAVSD NMFVHNNSKH GRRARRL-DP
MdCOE1       NPEMCRVLLT ---HEIMCSR CCDKKSCGNR NETPSDPVII D--RFFLKFF LKCNQNCLKN AGNPRDMRR- FQVVVSTTVN VDGHVLAVSD NMFVHNNSKH GRRARRL-DP
OaCOE1       XXXXXXXXXX XXXXXXXXXX XXXXXXXXXX XXXXXXXXXX XXXXXXXXXX XXXXXXXXXX XXXXXXXXXX XXXXXXXXXX XXXXXXXXXX XXXXXXXXXX XXXXXXXXXX
GgCOE1       NPEMCRVLLT ---HEIMCSR CCDKKSCGNR NETPSDPVII D--RFFLKFF LKCNQNCLKN AGNPRDMRR- FQVVVSTTVN VDGHVLAVSD NMFVHNNSKH GRRARRL-DP
XtCOE1       XXXXXXXXXX XXXXXXXXXX XXXXXXXXXX XXXXXXXXXX XXXXFFLKFF LKCNQNCLKN AGNPRDMRR- FQVVVSTTIN VDGHVLAVSD NMFVHNNSKH GRRARRL-DP
DrCOE1       NPEMLTALFL SSSPSVSISR CCDKKSCGNR NETPSDPVII D--RFFLKFF LKCNQNCLKN AGNPRDMRR- FQVVVSTTVS VDGHVLAVSD NMFVHNNSKH GRRARRL-DP
COE2
MmCOE2       NPEMCRVLLT ---HEVMCSR CCEKKSCGNR NETPSDPVII D--RFFLKFF LKCNQNCLKT AGNPRDMRR- FQVVLSTTVN VDGHVLAVSD NMFVHNNSKH GRRARRL-DP
HsCOE2       NPEMCRVLLT ---HEVMCSR CCEKKSCGNR NETPSDPVII D--RFFLKFF LKCNQNCLKT AGNPRDMRR- FQVVLSTTVN VDGHVLAVSD NMFVHNNSKH GRRARRL-DP
OaCOE2       XXXXXXXXXX XXXXXXXXXX XXXXXXXXXX XXXXXXXXXX XXXXXXXXXX XXXXXXXXXX XXXXXXXRK- VQVTGASKVN VDGHVLAVSD NMFVHNNSKH GRRARRL-DP
GgCOE2       NPEMCRVLLT ---HEVMCSR CCEKKSCGNR NETPSDPVII DSYRFFLKFF LKCNQNCLKT AGNPRDMRR- FQVVLSTTVN VDGHVLAVSD NMFVHNNSKH GRRARRL-DP
XtCOE2       NPEMCRVLLT ---HEVMCSR CCEKKSCGNR NETPSDPVII D--RFFLKFF LKCNQNCLKT AGNPRDMRR- FQVVLSTTVN VDGHVLAVSD NMFVHNNSKH GRRARRL-DP
DrCOE2       NPEMCRVLLT ---HEVMCSR CCEKKSCGNR NETPSDPVII D--RFFLKFF LKCNQNCLKT AGNPRDMRR- FQVVLSTTVC VDGPVLAISD NMFVHNNSKH GRRSRRM-DP
COE3
MmCOE3       NPEMCRVLLT ---HEIMCSR CCDKKSCGNR NETPSDPVII D--RFFLKFF LKCNQNCLKN AGNPRDMRR- FQVVVSTTVN VDGHVLAVSD NMFVHNNSKH GRRARRL-DP
HsCOE3       NPEMCRVLLT ---HEIMCSR CCDKKSCGNR NETPSDPVII D--RFFLKFF LKCNQNCLKN AGNPRDMRR- FQVVVSTTVN VDGHVLAVSD NMFVHNNSKH GRRARRL-DP
OaCOE3       NPEMCRVLLT ---HEIMCSR CCDKKSCGNR NETPSDPVII D--RFFLKFF LKCNQNCLKN AGNPRDMRR- FQVVVSTTVN VDGHVLAVSD NMFVHNNSKH GRRARRL-DP
GgCOE3       XXXXXXXXXX XXXXXXXXXX CCDKKSCGNR NETPSDPVII D--RFFLKFF LKCNQNCLKN AGNPRDMRR- FQVVVSTTVN VDGHVLAVSD NMFVHNNSKH GRRARRL-DP
XtCOE3       NPEMCRVLLT ---HEIMCSR CCDKKSCGNR NETPSDPVII D--RFFLKFF LKCNQNCLKN AGNPRDMRR- FQVVVSTTVN VDGHVLAVSD NMFVHNNSKH GRRARRL-DP
DrCOE3       NPEMCRVLLT ---HEIMCSR CCDKKSCGNR NETPSDPVII D--RFFLKFF LKCNQNCLKN AGNPRDMRR- FQVVVSTTVN VDGHVLAVSD NMFVHNNSKH GRRARRL-DP
EBF4
MmEBF4       NPEMCRVLLT ---HEIMCSR CCDRKSCGNR NETPSDPVII D--RFFLKFF LKCNQNCLKN AGNPRDMRR- FQVVVSTTVS VDGHVLAVSD NMFVHNNSKH GRRARRL-DP
HsEBF4       NPEMCRVLLT ---HEIMCSR CCDRKSCGNR NETPSDPVII D--RFFLKFF LKCNQNCLKN AGNPRDMRR- FQVVVSTTVS VDGHVLAVSD NMFVHNNSKH GRRARRL-DP
MdEBF4       NPEMCRVLLT ---HEIMCSR CCDKKSCGNR NETPSDPVII D--RFFLKFF LKCNQNCLKN AGNPRDMRR- FQVVVSTTVS VDGHVLAVSD NMFVHNNSKH GRRARRL-DP
OaEBF4       NPEMCRVLLT ---HEIMCSR CCDKKSCGNR NETPSDPVII D--RFFLKFF LKCNQNCLKN AGNPRDMRR- FQXXXXXXXX XXXXXXXXXX XXXXXXXXXX XXXXXXXXXX
UNASSIGNED
DrCOE        NPEMCRVLLT ---HETMCSR CCDKKSCGNR NETPSDPVII D--RFFLKFF LKCNQNCLKN AGNPRDTRR- FQVLVSTTAS VKAHILAISD NIFVHNNSKH GRRARRF-EL

LAMPREYS
Pm COE-A     XXXXXXXXXX XXXXXXXXSR CCDKKNCGNR NETPSDPVII D--RFFLKFF LKCNQNCLKN AGNPRDMRR- FQVVVSTTVH VDGHVLAVSD NMFVHNNSKH GRRARRI-DP
Pm COE-B     XXXXXXXXXX XXXXXXXXXX XXXXXXXXXX XXXXXXXXXX XXXXXXXXXX XXXXXXXXXX XXXXXXXXXX XXXXXXXXXX XXXXXXXXXX XXXXXXXXXX XXXXXXXXXX

NON-VERTEBRATES
CiCOE        NPEMRRVLLT ---HEIMCSR CCDKKSCGNR NETPSDPVVI D--RYFLKFF LKCNQNCLKN AGNPRDMRR- FQVVVSTTVH VDGHVLAVSD NMFVHNNSKH GRRARRV-DP
BfCOE        NPEMQRVLLT ---HEIMCSR CCDKKSCGNR NETPSDPVII D--RFFLKAF LKCNQNCLKN AGNPRDMRR- FQVVIATTVN VDGHVLAVSD NMFVHNNSKH GRRARRL-DP
SpCOE        NPEMCRVLLT ---HEIMCSR CCDKKSCGNR NETPSDPVII D--RFFLKFF LKCNQNCLKN AGNPRDMRR- FQVVISTTPN VDGHVLAMSD NMFVHNNSKH GRRARRL-DP
LgCOE        NPEMCRVLLT ---HEVMCSR CCDKKSCGNR NETPSDPVII D--RFFLKFF MKCNQNCLKN AGNPRDMRR- FQVAVSTSVA IDGPLLTISD NMFVHNNSKH GRRARRL-DP
CcCOE        NPEMCRCLLT ---HEIMCSR CCDKKSCGNR NETPSDPVII D--RFFLKFF MKCNQNCLKN AGNPRDMRR- FQVSIGTSSS VEGPLLAVSD NMFVHNNSKH GRRARRL-DP
DmCOE        NPEMCRVLLT ---HEVMCSR CCDKKSCGNR NETPSDPVII D--RFFLKFF LKCNQNCLKN AGNPRDMRR- FQVVISTQVA VDGPLLAISD NMFVHNNSKH GRRAKRL-DT
CeCOE        NPEMCRVLLT ---HEVMCSR CCEKKSCGNR NETPSDPVII D--RFFLKFF LKCNQNCLKN AGNPRDMRR- FQVVLCSSAR IDGPLLAVSD NMFVHNNSKH GRRTKRT-DA
NvCOE        NPEMRRVLLT ---HEVMCSR CCEKKSCGNK NETPSDPVVV D--RYCLKFF MKCNQNCLKN AGNPKDMRR- FQVHVSTSVD PMYGMIACSD NMFVHNNSKH GRRTRSRADG
AmCOE        NPDFRRVLLT ---HELICSR CLERRSCGNK NDTPSDPIIL D--NFRLKIF AKCNQNCLKN AGNPKDSRRR FQVALYEIDK VSGQPIACSD SMFVHNNSKH GRRPIYR-DN
```

Ig-like/Plexin/Transcription Factor Domain

```
GNATHOSTOMES
COE1
MmCOE1       SEGT-PSYLE H--------- ---------- -ATPCIKAIS PSEGWTTGGA TVIIIGDNFF DGLQVIFGTM L-VWSE---L ITPHAIRVQT PPRHIPGVVE VTLSYKSK--
HsCOE1       SEGT-PSYLE H--------- ---------- -ATPCIKAIS PSEGWTTGGA TVIIIGDNFF DGLQVIFGTM L-VWSE---L ITPHAIRVQT PPRHIPGVVE VTLSYKSK--
MdCOE1       SEGT-PSYLE H--------- ---------- -ATPCIKAIS PSEGWTTGGA TVIIIGDNFF DGLQVIFGTM L-VWSE---L ITPHAIRVQT PPRHIPGVVE VTLSYKSK--
OaCOE1       XXXXXXXXXX XXXXXXXXXX XXXXXXXXXX XXXXXXXXXX XXXXXXXXXX XXXXXXXXXX XXXXXXXXXX XXXXXXXXXL ITPHAIRVQT PPRHIPGVVE VTLSYKSK--
GgCOE1       SEGT-PSYLE H--------- ---------- -ATPCIKAIS PSEGWTTGGA TVIIIGDNFF DGLQVIFGTM L-VWSE---L ITPHAIRVQT PPRHIPGVVE VTLSYKSK--
XtCOE1       SDGT-PSYLE HEA------- ---------- -ATPCIKAIS PSEGWTTGGA TVIVIGDNFF DGLQVIFGTM L-VWSE---L ITPHAIRVQT PPRHIPGVVE VTLSYKSK--
DrCOE1       SEGT-PSYLE H--------- ---------- -ATPCIKAIS PSEGWTTGGA TVIIIGDNFF DGLQVIFGTM L-VWSE---L ITPHAIRVQT PPRHIPGVVE VTLSYKSK--
COE2
MmCOE2       SE-------- ---------- ---------- -ATPCIKAIS PSEGWTTGGA MVIIIGDNFF DGLQVVFGTM L-VWSE---L ITPHAIRVQT PPRHIPGVVE VTLSYKSK--
HsCOE2       SE-------- ---------- ---------- -ATPCIKAIS PSEGWTTGGA MVIIIGDNFF DGLQVVFGTM L-VWSE---L ITPHAIRVQT PPRHIPGVVE VTLSYKSK--
OaCOE2       SE-------- ---------- ---------- -ATPCIKAIS PSEGWTTGGA MVIIIGDNFF DGLQVVFGTM L-VWSE---L ITPHAIRVQT PPRHIPGVVE VTLSYKSK--
GgCOE2       SE-------- ---------- ---------- -ATPCIKAIS PSEGWTTGGA MVIIIGDNFF DGLQVVFGTM L-VWSE---L ITPHAIRVQT PPRHIPGVVE VTLSYKSK--
XtCOE2       SE-------- ---------- ---------- -ATPCIKAIS PSEGWTTGGA MVIIIGDNFF DGLQVVFGTM L-VWSE---L ITPHAIRVQT PPRHIPGVVE VTLSYKSK--
DrCOE2       NETV-ENNME Y--------- ---------- -ATPCIKAIS PSEGWTTGGA MVIVIGENFF DGLQVVFGSM L-VWSE---L ITPHAIRVQT PPRHIPGVVE VTLSYKSK--
COE3
MmCOE3       SE-------- ---------- ---------- -ATPCIKAIS PSEGWTTGGA TVIIIGDNFF DGLQVVFGTM L-VWSE---L ITPHAIRVQT PPRHIPGVVE VTLSYKSK--
HsCOE3       SEGTAPSYLE N--------- ---------- -ATPCIKAIS PSEGWTTGGA TVIIIGDNFF DGLQVVFGTM L-VWSE---L ITPHAIRVQT PPRHIPGVVE VTLSYKSK--
OaCOE3       SEXXXXXXXX XXXXXXXXXX XXXXXXXXXX XXXXXXXXXX XXXXXXXXXX XXXXXXXXXX XXXXXXXXXX XXXXXXXXXX XXXXXXXXXX XXXXXXXXXX XXXXXXXXXX
GgCOE3       SEGTAPSYLE NA-------- ---------- -ATPCIKAIS PSEGWTTGGA TVIIIGDNFF DGLQVVFGTM L-VWSE---L ITPHAIRVQT PPRHIPGVVE VTLSYKSK--
XtCOE3       SEGTAPSYLE NV-------- ---------- -ATPCIKAIS PSEGWTTGGA TVIIIGDNFF DGLQVVFGTM L-VWSE---L ITPHAIRVQT PPRHIPGVVE VTLSYKSK--
DrCOE3       SEGTATPYLE NA-------- ---------- -ATPCIKAIS PSEGWTTGGA TVIIIGDNFF DGLQVVFGTM L-VWSE---L ITPHAIRVQT PPRHIPGVVE VTLSYKSK--
EBF4
MmEBF4       SEA------- ---------- ---------- -ATPCIKAIS PGEGWTTGGA TVIIIGDNFF DGLQVVFGNV L-LWSE---L ITPHAIRVQT PPRHIPGVVE VTLSYKSK--
HsEBF4       SEA------- ---------- ---------- -ATPCIKAIS PGEGWTTGGA TVIVIGDNFF DGLQVVFGNV L-VWSE---L ITPHAIRVQT PPRHIPGVVE VTLSYKSK--
MdEBF4       SEA------- ---------- ---------- -ATPCIKAIS PSEGWTTGGA TVIVIGDNFF DGLQVVFGTM L-VWSE---L ITPHAIRVQT PPRHIPGVVE VTLSYKSK--
OaEBF4       XXXXXXXXXX XXXXXXXXXX XXXXXXXXXX XXXXXXXXXX XXXXXXXXXX XXXXXXXXXX XXXXXXXXXX XXXXXXXXXX XXXXXXXXXX XXXXXXXXXX XXXXXXXXXX
UNASSIGNED
DrCOE        IEA------- ---------- ---------- -EIPCIKAIS PSEGWTSGGA SVIIIGDQFF SGLQVVFGTM L-VWSE---V ITPHAIRVQT PPRHIPGVVE VTLSYKSK--

LAMPREYS
Pm COE-A     SE-------- ---------- ---------- -ATPCIKAIS PSEGWTTGGA TVIIIGDNFF DGLQVVFGTM L-VWSE---L ITPHAIRVQT PPRHIPGVVE VTLSYKSK--
Pm COE-B     XXXXXXXXXX XXXXXXXXXX XXXXXXXXXX XXXXXXXXXX XXXXXXXXXX XXXXXXXXXX XXXXXXXXXX XXXXXXXXXX XXXXXXXXXX XXXXXXXXXX XXXXXXXXXX

NON-VERTEBRATES
CiCOE        SEGNFFFIES ---------- ---------- -ASPTIKAIN PAEGWTTGGA TVVIVGENFF DGLQVVFGSM V-VWSE---L ITQHAIRVQT PPRHLPGVVE VTLSYKNK--
BfCOE        SEDGEMSFCG DSPNINRGQK ---------- -ATPCIKAIS PSEGWTTGGA TVIIIGDNFF DGLQVVFGTM L-VWSE---L ITPHAIRVQT PPRHIPGVVE VTLSYKSK--
SpCOE        SEG------- ---------- ---------- -ATPCIKAIS PSEGWTTGGA TVIIVGDNFF DGLQVVFGSM I-VWSE---L ITPHAIRVQT PPRHIPGVVE VTLSYKSK--
LgCOE        TEGGDLRFSH LRPKLSPFAA ---------- -TTPCIKAIC PSEGWTSGGT TVIIIGDNFF DGLQLVFGTM L-VWSE---L LTSHAIRVQT PPRHIPGVVE VTLSYKSK--
CcCOE        GDAGFLCA-- ---------- ---------- -ATPTIKAIC PSEGWTTGGT TVIIIGDNFF DGLQVVFGTM L-VWSE---L ITSHAIRVQT PPRHIPGVVE VTLSYKSK--
DmCOE        TEGTGNTSLS ISGHPLAPDS TYDGLYPPLP VATPCIKAIS PSEGWTTGGA TVIIVGDNFF DGLQVVFGTM L-VWSE---L ITSHAIRVQT PPRHIPGVVE VTLSYKSK--
CeCOE        SDDSEYSESA ELPS------ ---------- -SVPVIKALF PSEGWIQGGT QVVLIGENFF EGLQVAFGTA SPNWGESVQL ISPHAIRVTT PPKHSAGPVD VTLQYKSK--
NvCOE        NEQDTSA--- ---------- ---------- -DDPCIKAIC PNEGWTIGGS NVILIGDNFF DGLQVVFGSF I-VWSE---F ITPHALRVQA PPSPMPGVVK VYLMHKEK--
AmCOE        VVGD------ ---------- ---------- -GKPCIVSIY PNEGWTTGGS RITVIGVNFF EGLDIVFGTV P-VQSE---V LSPNAIAVRT PPATMTGEVD VTLIFRASGA
```

```
                              HELIX H1          Linker         HELIX H2d          HELIX H2A
GNATHOSTOMES
COE1
MmCOE1        QFCKGTPGRF IYT-ALNEPT IDYGFQRLQK VIPRHPGDPE RLPKEVILKR AADLVEALYG MPHNNQ--EI ILKRAADIAE ALYSVPRNHN
HsCOE1        QFCKGTPGRF IYT-ALNEPT IDYGFQRLQK VIPRHPGDPE RLPKEVILKR AADLVEALYG MPHNNQ--EI ILKRAADIAE ALYSVPRNHN
MdCOE1        QFCKGTPGRF IYT-ALNEPT IDYGFQRLQK VIPRHPGDPE RLPKEVILKR AADLVEALYG MPHNNQ--EI ILKRAADIAE ALYSVPRNHN
OaCOE1        QFCKGTPGRF IYT-ALNEPT IDYGFQRLQK VIPRHPGDPE RLPKEVILKR AADLVEALYG MPHNNQ--EI ILKRAADIAE ALYSVPRNHN
GgCOE1        QFCKGTPGRF IYT-ALNEPT IDYGFQRLQK VIPRHPGDPE RLPKEVILKR AADLVEALYG MPHNNQ--EI ILKRAADIAE ALYSVPRNHN
XtCOE1        QFCKGTPGRF IYT-ALNEPT IDYGFQRLQK VIPRHPGDPE RLPKEVILKR AADLVEALYG MPHNNQ--EI ILKRAADIAE ALYSVPRNHS
DrCOE1        QFCKGTPGRF IYT-ALNEPT IDYGFQRLQK VIPRHPGDPE RLPKEVILKR AADLVEALYG MPHNNQ--EI ILKRAADIAE ALYNVPRGHN
COE2
MmCOE2        QFCKGAPGRF IYT-ALNEPT IDYGFQRLQK VIPRHPGDPE RLAKEMLLKR AADLVEALYG TPHNNQ--DI ILKRAADIAE ALYSVPRNPS
HsCOE2        QFCKGAPGRF IYT-ALNEPT IDYGFQRLQK VIPRHPGDPE RLAKEMLLKR AADLVEALYG TPHNNQ--DI ILKRAADIAE ALYSVPRNPS
OaCOE2        QFCKGAPGRF IYT--LNEPT IDYGFQRLQK VIPRHPGDPE RLAKEMLLKR AADLVEALYG TPHNNQ--DI ILKRAADIAE ALYSVPRNPS
GgCOE2        QFCKGAPGRF IYT-ALNEPT IDYGFQRLQK VIPRHPGDPE RLAKEMLLKR AADLVEALYG TPHNNQ--DI ILKRAADIAE ALYSVPRNPA
XtCOE2        QFCKGAPGRF IYT-ALNEPT IDYGFQRLQK VIPRHPGDPE RLAKEMLLKR AADLVEALYG TPHNNQ--DI ILKRAADIAE ALYSVPRNHN
DrCOE2        QFCKGAPGRF IYT-ALNEPT IDYGFQRLQK LIPRHPGDPD KLAKEMLLKR AADVVESLYG NTTSNQ--DM LLKRAADIAE ALYSVPRPHS
COE3
MmCOE3        QFCKGAPGRF VYT-ALNEPT IDYGFQRLQK VIPRHPGDPE RLPKEVLLKR AADLVEALYG MPHNNQ--EI ILKRAADIAE ALYSVPRNHN
HsCOE3        QFCKGAPGRF VYT-ALNEPT IDYGFQRLQK VIPRHPGDPE RLPKEVLLKR AADLVEALYG MPHNNQ--EI ILKRAADIAE ALYSVPRNHN
OaCOE3        XXXXXXXXXX XXXXXXXXXX XXXXXXXXXX XXXXXXXXXX XXXXXXXXXX XXXXXXXXXX XXXXXXXXXX XXXXXXXXXX XXXXXXXXXX
GgCOE3        QFCKGAPGRF VYT-ALNEPT IDYGFQRLQK VIPRHPGDPE RLPKEVLLKR AADLVEALYG MPHNNQ--EI ILKRAADIAE ALYSVPRNHN
XtCOE3        QFCKGAPGRF VYT-ALNEPT IDYGFQRLQK VIPRHPGDPE RLPKEVLLKR AADLVEALYG MPHNNQAKEI ILKRAADIAE ALYSVPRNHN
DrCOE3        QFCKGAPGRF VYT-ALNEPT IDYGFQRLQK VIPRHPGDPE RLPKEVLLKR AADLVEALYG MPHNNQ--EI ILKRAADIAE ALYSVPRNHN
EBF4
MmEBF4        QFCKGAPGRF VYT-ALNEPT IDYGFQRLQK VIPRHPGDPE RLPKEVLLKR AADLAEALYG VPSSNQ--EL LLKRAADVAE ALYSAPRAPA
HsEBF4        QFCKGCPGRF VYT-ALNEPT IDYGFQRLQK VIPRHPGDPE RLPKEVLLKR AADLAEALYG VPGSNQ--EL LLKRAADVAE ALYSTPRAPG
MdEBF4        QFCKGAPGRF VYT-ALNEPT IDYGFQRLQK VIPRHPGDPE RLPKEVLLKR AADLVEALYG MPHSNQ--DL IIKRAADIAE ALYSVPRTPS
OaEBF4        XXXXXXXXXX XXXXXXXXXX XXXXXXXXXX XXXXXXXXXX XXXXXXXXXX XXXXXXXXXX XXXXXXXXXX XXXXXXXXXX XXXXXXXXXX
UNASSIGNED
DrCOE         QFCRGAPGRF VYT-ALNEPT IDYGFQRLQK VIPHHPGDVE RLPKEVLLKR AADLIETFYG APQNNQ--EI ILKRASDIAE ALNSIPRNPS

LAMPREYS
Pm COE-A      QFCKGAPGRF VYT-ALNEPT IDYGFQRLQK LIPRHPGDPE RLPK------ ---------- -----Q--EI ILKRAADIAE ALYSVPRNHN
Pm COE-B      XXXXXXXXXX XXXXALNEPT IDYGFQRLQK VIPRHPGDPE RLPKEVILKR AADLVEALYG LPHYNQ--EI ILKRAADIAE ALYSMPRHQN

NON-VERTEBRATES
CiCOE         QFCSGAPGRF VYT-ALNEPT LDYGFQRLLK TVPRHPGDPE RLPK------ ---------- --------EI ILKRAADVME AVISRQYAPP
BfCOE         QFCKGAPGRF VYT-SLNEPT IDYGFQRLAK LVPRHPGDPE RLPK------ ---------- --------EI ILKRAADLAE AIYSMPRNPN
SpCOE         QFCKGAPGRF VYV-SLTEPT IDYGFQRLSK LVPRHPGDPD RLPK------ ---------- --------EI ILKRAADLAE ALYSMPRN--
LgCOE         QFCKGAPGRF VYT-ALTEPT IDYGFQRLMK LVPRHPGDPE KLPK------ ---------- --------EI ILKRAADLAE ALYSMPRNHN
CcCOE         QFCKGAPGRF VYT-ALTEPT IDYGFQRLMK LVPRHPGDPE RLPK-GICEG HVGPLSSQIF L-SCPQ--EI ILKRAADLAE ALYSMPRNPN
DmCOE         QFCKGSPGRF VYVSALNEPT IDYGFQRLQK LIPRHPGDPE KLQK------ ---------- --------EI ILKRAADLVE ALYSMPRSPG
CeCOE         TYSRGTPLRF SYI-TLAEPG IEYGFQRLQK LLPKYPGDPE RLPK------ ---------- --------DQ ILKRAAELAE ALYNRTSTES
NvCOE         QYCKYAPAKF GYT-ALVEPT IDYGFQRLSK LIPRHPGDPE RIPK------ ---------- --------EI VLKRAADLAE TLYQMPRTPT
AmCOE         QFCISNPGKF LYT-APDDQI FENSFCRIER VI-RQQDDPD QLPK------ ---------- --------DL VLQRAAELLE SCFLSSRGA-
```

Figure S2 : Alignment of the DBD, IPT and HLH domains of the COE proteins. Species are indicated on the top lines with abbreviations as in Fig. 1. Additional species abbreviations : Oa, *Ornithorhynchus anatinus*; Gg, *Gallus gallus*; Sp, *Strongylocentrotus purpuratus*; Ce, *Caenorhabditis elegans*; Am, *Amphimedon queenslandica.* Highly truncated (e.g., XtCOE1, PmCOE-B, OaCOE1, OaCOE3, OaEBF4) or divergent (CeCOE, AmCOE) predicted sequences were included in this alignment. Dashes indicate missing residues. X stands for undetermined sequences. The positions correspond to the DBD, IPT, Helix 1, H2d and H2a domains are shaded in orange, yellow, green, pale blue and dark blue, respectively. The linker separating H1 and H2 is squared.
