# Supplementary material for: The metazoan history of the COE transcription factors. Selection of a variant HLH motif by mandatory inclusion of a duplicated exon in vertebrates
Source: BMC Evol Biol. 2008 May 2;8:131. doi: 10.1186/1471-2148-8-131 (PMC2394523; doi:10.1186/1471-2148-8-131)
Supplement: Additional file 3 — Alignment of the DBD, IPT and HLH domains of the COE proteins. [file 1471-2148-8-131-S3.pdf]

```
                            Linker                    Helix 2d                    Helix 2a

PmCOE-B scaffold-569 CCGAGACACCCCGGCGACCCCGAGAGGCTACCCAAGGAGGTGATCTTGAAGCGCGGCAGATTTAGTGGAGGCTTTGTACGGCCTGCCTCATTACAACCAGGAAATCATTCTCAAGAGAGCCGCGGACATCGCCGAAGCT

LfCOE-B EST A13YL15  CCGAGACACCCCGGCGACCCCGAGAGGCTACCCAAGGAGGTGATCTTGAAGCGCGGCAGATTTAGTGGAGGCTTTGTACGGCCTGCCTCATTACAACCAGGAAATCATTCTGAAGAGAGCCGCCGACATCGCCGAAGCT
LfCOE-B EST A26YP16  CCGAGACACCCCGGCGACCCCGAGAGGCTACCCAAG----------------------------------------------------------------GAAATCATTCTGAAGAGAGCCGCCGACATCGCCGAAGCT

LfCOE-B/PmCOE-         P R H P G D P E R L P K E V I L K R A A D L V E A L Y G L P H Y N Q E I I L K R A A D I A E A
```

Structure of *P. marinus* and *L. fluviatilis* COE-B transcripts at the level of the H2d coding region. The alignment is restricted to the sequence covering the linker, H2d and N-terminal part of H2a of lamprey COE-B proteins. The upper line shows *PmCOE-B* mRNA sequence, as predicted from the analysis of *P. marinus* genomic scaffold 569. Arrowheads indicate the positions of introns. The lower lines show partial *L . fluviatilis* COE-B EST sequences (A13YL15 and A26YP16), obtained in a large-scale EST sequencing project currently in progress. The deduced amino acid sequence is identical in both species and given below the alignment, using the same colour code is as in Fig. 2C. The two *L. fluviatilis* ESTs differ by the presence of Helix 2d coding region, thus providing evidence for alternative splicing in the lamprey.
