# Supplementary material for: The metazoan history of the COE transcription factors. Selection of a variant HLH motif by mandatory inclusion of a duplicated exon in vertebrates
Source: BMC Evol Biol. 2008 May 2;8:131. doi: 10.1186/1471-2148-8-131 (PMC2394523; doi:10.1186/1471-2148-8-131)
Supplement: Additional file 4 — Structure of P. marinus and L. fluviatilis COE-B transcripts at the level of the H2d coding region. [file 1471-2148-8-131-S4.pdf]

```
EBF/MmCOE1                    1 MFGIQESIQRSGSSMKEEPLGSGMNAVRTWMQGAGVLDANTAAQSGVGLA
Col/DmCOE   1 MEWGRKLYPSAVSGPRSAGGLMFGLPPTAAVDMNQPRGPMTSLKEEPLGSRWAMQPVVDQSNLGIG

RAHFEKQPPSNLRKSNFFHFVLALYDRQGQPVEIERTAFVGFVEKEKEANSEKTNNGIHYRIQLLYSNGIRTEQDFYVRL
RAHFEKQPPSNLRKSNFFHFVIALYDRAGQPIEIERTAFIGFIEKDSESDATKTNNGIQYRLQLLYANGARQEQDIFVRL

IDSMTKQAIVYEGQDKNPEMCRVLLTHEIMCSRCCDKKSCGNRNETPSDPVIIDRFFLKFFLKCNQNCLKNAGNPRDMRR
IDSVTKQAIIYEGQDKNPEMCRVLLTHEVMCSRCCDKKSCGNRNETPSDPVIIDRFFLKFFLKCNQNCLKNAGNPRDMRR

FQVVVSTTVNVDGHVLAAPDNMFVHNNSKHGRRARRLDPSEGT---------------PSYLEH------ATPCIKAISP
FQVVISTQVAVDGPLLAISDNMFVHNNSKHGRRAKRLDTTEGTGNTSLSISGHPLAPDSTYDGLYPPLPVATPCIKAISP

SEGWTTGGATVIIIGDNFFDGLQVIFGTMLVWSELTGPHSIRVQTPPRHIPGVVEVTLSYKSKQFCKGTPGRFIY
SEGWTTGGATVIIVGDNFFDGLQVVFGTMLVWSELITSHAIRVQTPPRHIPGVVEVTLSYKSKQFCKGSPGRFVY

     Helix 1           Linker        Helix 2d             Helix 2a
-TALNEPTIDYGFQRLQKVIPRHPGDPERLPKEVILKRAADLVEALYGMPHNNQEIILKRAADIAEALYSVPRNHNQLPA
VSALNEPTIDYGFQRLQKLIPRHPGDPEKLQ----------------------KEIILKRAADLVEALYSMPRSPGGSTG

 ----Col/EBFΔH1-----                                 ------ColΔH2--------
                    --ColΔLH2----------------------------------------------
                          --EBFΔ370-383--

  EBF* (1-429)
LANTSVHAGMMGVNSFSGQLAVNVSEASQATNQGFTRNSSSVSPHGYVPSTTPQQTNYNSVTTSMNGYGSAAMSNLGGSP
FNSYSGQLAVSVQDGSGQWTEDDYQRAQSSSVSPRGGYCSSASTPHSSGGSYGATAASAAVAATANGYAPAPNMGTLSSS
     Col* (1-449)

TFLNGSAANSPYAIVPSSPTMASSTSLPSNCSSSSGIFSFSPANMVSAVKQKSAFAPVVRPQTSPPPTCTSTNGNSLQAI
PGSVFNSTSMSAVSSTWHQAFVQHHHAATAHPHHHYPHPHQPWHNPAVSAATAAAV  557

SGMIVPPM 591
```

Figure S4

Diagrammatic alignment of EBF (top) and Col protein amino-acid sequences. Conserved residues in the DBD (red), IPT (yellow) and HLH (italics) domains, including the H1-H2 linker (green) are in bold. The extent of the H1 and H2 deletions introduced in the modified versions of EBF and Col (Fig. 2) are shown by dashed lines. The position of the EBF internal deletion (residues 370 to 383) described in Hagman et al., 1995 is also indicated. In frame Methionine initiator codons are underlined. The position of the C-terminal valine of truncated EBF* and Col* (Fig. 2) is indicated by a star. Position of the putative zinc finger (Hagman et al., 1995) in the DBD is red underlined.
