# Supplementary material for: The metazoan history of the COE transcription factors. Selection of a variant HLH motif by mandatory inclusion of a duplicated exon in vertebrates
Source: BMC Evol Biol. 2008 May 2;8:131. doi: 10.1186/1471-2148-8-131 (PMC2394523; doi:10.1186/1471-2148-8-131)
Supplement: Additional file 5 — Diagrammatic alignment of EBF and Col protein amino-acid sequences. The sequence alignment provided shows the position of the H1 and H2 deletions introduced in modified versions of EBF and Col used in DNA-binding assays (Fig. 3) and EBF internal deletion described in Hagman et al., 1995 [17]. [file 1471-2148-8-131-S5.pdf]

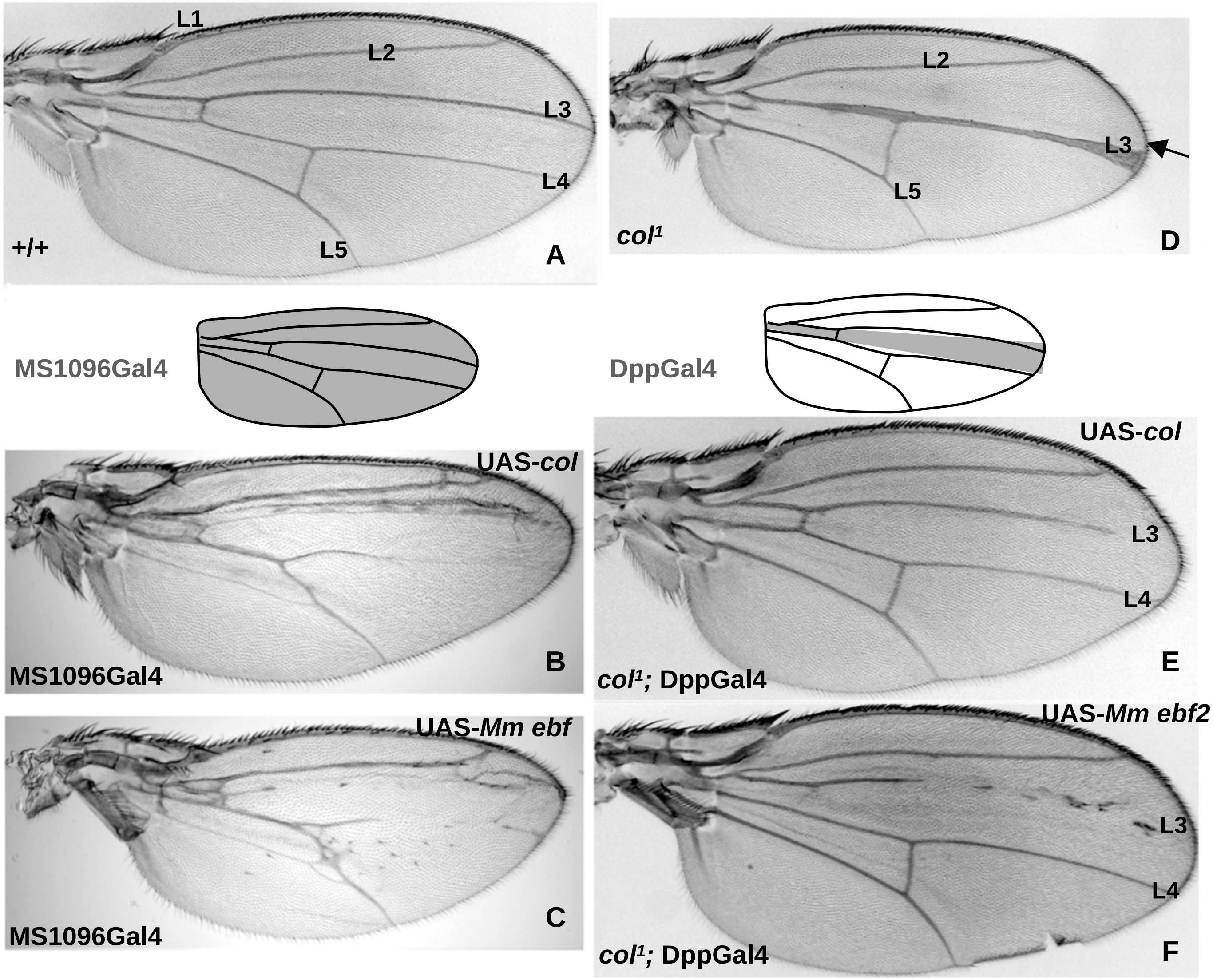

EBF can substitute for Col in patterning of the *Drosophila* wing. Adult wings, with anterior up and distal right. (A) wt; L1-L5 indicate the longitudinal veins. (B, C) MS1096Gal4-driven expression of *col*, (B) and *Mm ebf*, (C) : Ubiquitous expression of either Col or EBF in the wing pouch results in a nearly complete transformation of the wing into intervein tissue. (D) *col*^{1} mutant wing : a central larger L3 vein is present, while the L3-L4 intervein and vein L4 are missing. (E, F**)**, rescue of the *col*^{1} mutant phenotype by DppGal4-driven expression of either *col* (E) or *Mm ebf2* (F). In both cases, there is complete rescue both of L3-L4 intervein and L4 vein formation. Similar results were obtained with *Mm ebf1* in place of *Mm ebf2* (not shown). *Dpp-Gal4* expression results in a stripe of Col expression that is slightly larger anteriorly, compared to wild type, with an occasional loss of the distal-most part of vein L3 [48]. In *UAS-Mm ebf/*DppGal4 wings, supernumerary bristles form at the position of distal L3. Wing schemes show where MS1096Gal4 and DppGal4 drive expression.
