# Supplementary material for: The metazoan history of the COE transcription factors. Selection of a variant HLH motif by mandatory inclusion of a duplicated exon in vertebrates
Source: BMC Evol Biol. 2008 May 2;8:131. doi: 10.1186/1471-2148-8-131 (PMC2394523; doi:10.1186/1471-2148-8-131)
Supplement: Additional file 6 — EBF can substitute for Col in patterning of the Drosophila wing. This figure compares the phenotypes of adult Drosophila wings subject to targeted expression of either mouse EBF1 or EBF2 or Drosophila Col. [file 1471-2148-8-131-S6.pdf]

Table S1

	COE1	COE2	COE3	COE4	Unassigned
Mouse	**ENSMUSG00000057098**	**ENSMUSG00000022053**	**ENSMUSG00000010476**	**ENSMUSG00000053552**	_
Human	**ENSG00000164330**	**ENSG00000134025**	**ENSG00000108001**	**ENSG00000088881**	_
M. domestica	**ENSMODG00000008198**	_	_	**ENSMODG00000005256**	_
O. anatimus	**ENSOANG00000012338**	**ENSOANG00000004212**	**ENSOANG00000012326**	**ENSOANG00000005673**	
G. gallus	**ENSGALG00000003699**	**ENSGALG00000000242**	**ENSGALG00000010461**		
X. tropicalis	**ENSXETG00000001493**	**gi 38648990**	**ENSXETG00000011309**	_	_
D. rerio	**ENSDARG00000010608**	**gi 18858451**	**ENSDARG00000003613**	_	**ENSDARG00000016116**
P. marinus	_	_	_	_	**PmCOEA : Scaffolds-61646-2155-7544-27354-19799-42307 PmCOEB : Scaffold-569**
C. intestinalis	_	_	_	_	**ENSCINP00000006566**
B. floridae	_	_	_	_	**gi 45752204**
S. purpuratus	_	_	_	_	**Modification of gi115905857 (below)**
D. melanogaster	_	_	_	_	**CG10197**
C. elegans	_	_	_	_	**Y16B4A.1**
L. gigantae	_	_	_	_	**jgi Lotgi1 225788 estExt Genewise1Plus C sca 1500019**
C. capitata	_	_	_	_	**jgi Capca1 93575 e gw1 31 157 1**
N. vectensis					**jgi Nemve1 164975**
A. queenslandica	_	_	_	_	**Amq16**

Identification of the metazoan COE sequences used in this study

COE genes included in this study are identified by their Genbank accession numbers or Ensembl identification numbers when available. In the lamprey *P. marinus*, the scaffolds containing COE sequences are indicated by their number in the current Pre-Ensembl genome version. In *A. queenslandica*, the sequence used is the Geneid prediction, reported by Simionato et al. 2007. BMC Evol. Biol. 7, 33. All the gnathostome sequences could be assigned to one of the four COE1-4 classes and are thus indicated in the corresponding column, except one zebrafish sequence, included in the last column (unassigned sequences). Similarly, the lamprey and non-vertebrate sequences, which do not show any relationship to either of the gnathostome classes, are identified in the last column. The sea urchin *S. purpuratus* used in this study (see below) was manually predicted : it can be readily aligned with other deuterostome COE proteins over the whole length of the protein (similarities underlined below), unlike the automated prediction gi115905857, which appears erroneous in the C-terminal part of the molecule. The sea urchin *S. purpuratus* COE sequence used in this study was manually predicted at the level of the linker-Helix1-Helix2 domains, which leads to a modification of the automated prediction gi115905857. The novel predicted protein sequence is (with successive exons shaded in gray and yellow):

atgttcgggatccaggattcattgacgaggggttctaccaacctcaaagaggaaccaata

M F G I Q D S L T R G S T N L K E E P I

atcacggcggaggtcggatcggctgccgttagagtcggctggatgcagccaaccatggtg

I T A E V G S A A V R V G W M Q P T M V

gatcaaagtgccagttcgtgtgggatggcccgagcacacttcgagaaacaacccccgagt

D Q S A **S** S C G M A R A H F E K Q P P S

aatttacgaaagagtaacttcttccatttcgtgatcgcgctctacgaccgagcaggtcaa

N L R K S N F F H F V I A L Y D R A G Q

cccatcgaagtcgagaggacatccttcgttgatttcatcgaaaaagaaagggagccagac

P I E V E R T S F V D F I E K E R E P D

gctacgaagacaaacaatggcattcactaccgtatacaaatgttatttcacaatggagtg

A T K T N N G I H Y R I Q M L F H N **G** V

cgaacggagcaggatctctatgtgcgacttatcgactctatgacgaaacaggccatcata

R T E Q D L Y V R L I D S M T K Q A I I

tatgaaggacaggacaagaatccagagatgtgtcgtgtgctactcactcacgaaatcatg

Y E G Q D K N P E M C R V L L T H E I M

tgcagccgctgctgtgacaagaaaagctgtggcaatagaaacgagactccatctgatccg

C **S** R C C D K K S C G N R N E T P S D P

gtaatcatcgacagatttttcctcaagttcttcctcaagtgcaaccagaactgcttgaaa

V I I D **R** F F L K F F L K C N Q N C L K

aatgcagggaatccaagagatatgcgtcgatttcaggtggttatatctacaactccaaac

N A G N P R D M R R F Q V V I S T T P N

gttgatggtcatgtgctggccatgtccgataacatgtttgtacacaacaactccaaacac

V D G H V L A M S D N M F V H N N S K H

ggccggagagcgagaagacttgacccatctgaaggagctacgccgtgcatcaaggcaatc

G R R A R R L D P S E G **A** T P C I K A I

agccctagcgaaggatggacaacagggggcgccactgtcatcatcgtcggcgacaacttc

S P S E G W T T G G A T V I I V G D N F

ttcgacggactccaggtcgtctttggcagcatgatcgtctggagtgagctcatcacccccc

F D G L Q V V F G S M I V W S E L I T P

cacgccatcagagtacaaaccccaccccgacacatccccggggtggtggaggtcacattg

H A I R V Q T P P R H I P G V V E V T L

tcgtacaagtccaagcagttctgcaagggggcgc**caggcagg**ttcgtctacgtctcactg

S Y K S K Q F C K G A P G R F V Y V S L

acagagccgacaatagattacgggttccaaagactgagcaagcttgtcccgcggcatcct

T E P T I D Y G F Q R L S K L V P R H P

ggcgatccagatagactgccaaag**gaaa**tcattctgaagcgagcagcggatctg

G D P D R L P **K** E I I L K R A A D L

gcagaggccttgtatagtatgccaaggaatcaactccccattgggccgccccgatcaccc

A E A L Y S M P R N Q L P I G P P R S P

gccctcaacaacagctccggtagtctagtacccatgaactctttcggtagccagctagaa

A L N N S S G S L V P M N S F G S Q L E

cacgaaagtgaccaacattcaggatggaattccagcacatctgctatcctcctcctcttg

H E S D Q H S **G** W N S S T S A I L L L L

tgcctattgcctcctgcaagccaagggaagtccaaagctgcgtcagcaaaagtgtccatt

C L L P P A S Q G K S K A A S A K V S I

tcttcagctctctcgagggttattcaatttcagaagacaggagggagtattcaagcattc

S S A L S R V I Q F Q K T G G S I Q A F

ctcgacaacacaaaagagaccaagagtcaaccgttccttctggcaattggcaattccaag

L D N T K E T K S Q P F L L A I G N S K

gcccgaatcaccaactacatgatcattgtggatcacaaggccattccatgttcggcacgc

A R I T N Y M I I V D H K A I P C S A R

acggtcgaagcagcagtagatttactcttcaagacccactttgtctttggcctgcagtac

T V E A A V D L L F K T H F V F G L Q Y

tgcttgtctctgcggcagttctggacttttgtccagactgccatttttgaaatcgatatc

C L S L R Q F W T F V Q T A I F E I D I

ggagtgtcccgggaaacaccaagagtatacgtgaaattagatcgaaattgttag

G V S R E T P R V Y V K L D R N C -
